# Aerosol mass and size-resolved metal content in urban Bangkok, Thailand

**DOI:** 10.1007/s11356-022-20806-w

**Published:** 2022-06-15

**Authors:** James C. Matthews, Panida Navasumrit, Matthew D. Wright, Krittinee Chaisatra, Chalida Chompoobut, Robert Arbon, M. Anwar H. Khan, Mathuros Ruchirawat, Dudley E. Shallcross

**Affiliations:** 1 School of Chemistry, University of Bristol, Cantock’s Close, Bristol, BS8 1TS UK; 2 Laboratory of Environmental Toxicology, Chulabhorn Research Institute, Bangkok, 10210 Thailand; 3 Jean Golding Institute, Royal Fort House, University of Bristol, Bristol, BS8 1UH UK; 4 Department of Chemistry, University of the Western Cape, Robert Sobukwe Road, Bellville, 7375 South Africa

**Keywords:** Air pollution, Ultrafine aerosols, Particulate matter, Toxic metals, Carcinogens, Automotive exhaust

## Abstract

Inhalable particulate matter (PM) is a health concern, and people living in large cities such as Bangkok are exposed to high concentrations. This exposure has been linked to respiratory and cardiac diseases and cancers of the lung and brain. Throughout 2018, PM was measured in northern Bangkok near a toll road (13.87°N, 100.58°E) covering all three seasons (cool, hot and rainy). PM_10_ was measured in 24- and 72-h samples. On selected dates aerodynamic size and mass distribution were measured as 3-day samples from a fixed 5th floor inlet. Particle number concentration was measured from the 5th floor inlet and in roadside survey measurements. There was a large fraction of particle number concentration in the sub-micron range, which showed the greatest variability compared with larger fractions. Metals associated with combustion sources were most found on the smaller size fraction of particles, which may have implications for associated adverse health outcomes because of the likely location of aerosol deposition in the distal airways of the lung. PM_10_ samples varied between 30 and 100 μg m^−3^, with highest concentrations in the cool season. The largest metal fractions present in the PM_10_ measurements were calcium, iron and magnesium during the hot season with average airborne concentrations of 13.2, 3.6 and 2.0 μg m^−3^, respectively. Copper, zinc, arsenic, selenium, molybdenum, cadmium, antimony and lead had large non-crustal sources. Principal component analysis (PCA) identified likely sources of the metals as crustal minerals, tailpipe exhaust and non-combustion traffic. A health risk analysis showed a higher risk of both carcinogenic and non-carcinogenic health effects in the drier seasons than the wet season due to ingestion of nickel, arsenic, cadmium and lead.

**Graphical abstract:**

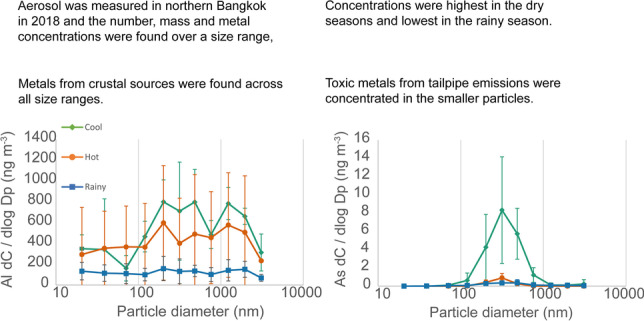

**Supplementary Information:**

The online version contains supplementary material available at 10.1007/s11356-022-20806-w.

## Introduction

Particulate matter (PM) is a growing health concern in many large cities and therefore knowledge of their formations and their physical and chemical properties is valuable. Analysis of the size distribution and metals present within the PM samples over different meteorological conditions can provide information about sources of particles as well as their likely health outcomes.

Since the 1990s, epidemiological evidence of mortality related to the inhalable fraction of PM has shown the need for further investigation into mechanistic explanations between particulate air quality and health outcomes (Dockery et al. 1993; Pope et al. 1995). Both outdoor air pollution as a whole, and specifically PM, have been classified as a class 1 carcinogen by the International Agency for Research on Cancer (IARC) (Loomis et al. 2013). An increased risk has been found related to traffic using nitrogen oxides (Ghosh et al. 2013), distance from roads (Houot et al. 2015) or density of petrochemical refuelling stations (Weng et al. 2009) as proxies for traffic exhaust, but a better understanding of the chemical content of PM is required to enable the causal agents of these effects to be determined and mitigated against. Toxicity of traffic emissions has largely concentrated on exhaust emissions (Atkinson et al. 2016) but also include emissions caused by abrasion (Selley et al. 2020).

PM is often measured as total mass < 10 μm in diameter, (PM_10_) and mass < 2.5 μm (PM_2.5_), and when inhaled can cause adverse health effects including respiratory and cardiac disease (Loomis et al. 2013). PM_2.5_ has been implicated with cancers of the lung (Raaschou-Nielsen et al. 2013) and the brain (Andersen et al. 2018); metallic particles found in the lung and brain are one possible cause (Maher et al. 2016; Raaschou-Nielsen et al. 2016). Smaller particles, sub-micron and ultrafine (< 100 nm), although less frequently measured, are receiving increasing attention due to their ability to penetrate deeper into the lung (Oberdörster 2000) and potentially translocate into the systemic circulation (Miller et al. 2017).

Some heavy metals (within which we forthwith include metalloids such as arsenic) are known carcinogens, and arsenic, cadmium, chromium and nickel are classified as either class 1 (known) or a class 2A (probable) carcinogen by IARC (Tchounwou et al. 2012) as oxidative stress can be a common toxicological result of exposure. Arsenic, derived from mining and found in ground water, is also used in batteries, the semiconductor industry and pigments. Cadmium is rare in the environment, but is used in batteries and electroplating, as well as being a component of paint in plastic products. Chromium is found in the Earth’s crust, but also used in many manufacturing processes including pigment production, leather tanning, smelting, roasting and extraction. Nickel is found in batteries, coins, as alloys, electroplating and stainless steel (Kim et al. 2015).

Within cities, a predominant source of PM is traffic, including exhaust tailpipe emissions, but also from abrasion including brake dust, tyre dust and resuspended particles from the ground. Within the combustion source, heavy metals can come from three sources: the fuel, the lubricant and engine wear. The mixture of metals within fuel and lubricants varied in samples across Europe but a general trend was that arsenic, mercury and selenium were found within the fuel and cadmium, chromium, copper, nickel, lead and zinc were found in the lubricants (Pulles et al. 2012).

In Bangkok, Thailand, exposure to pollutants has been associated with poor health outcomes in the exposed populations. Levels of benzene and polycyclic aromatic hydrocarbons are typically higher than in some European and American cities (Ruchirawat et al. 2005). Increased exposure to these pollutants has been shown to increase the risk of cancer within school children in Bangkok (Ruchirawat et al. 2007). PM_10_ aerosol has been analysed in Bangkok for metal content by Pongpiachan and Iijima (2016) from eight Pollution Control Department (PCD) sites, including 5 urban background and 3 roadside sites. Statistical analysis identified both crustal and vehicular sources of metals, identifying crustal metals (i.e. iron, aluminium, scandium, lanthanum, cerium and manganese) as the dominant source, vehicle tailpipe emissions as a second source (vanadium, cobalt, copper, lead, selenium and zinc) and a third source containing barium that is indicative of brake wear (non-combustion) vehicle emissions. Barium is well correlated with iron, copper and antimony in London measurements, and barium is considered a good tracer for brake dust (Gietl et al. 2010). The predominant source of antimony in global dust is brake wear, accounting for over half of global emissions (Zhu et al. 2020). Zhu et al. (2020) identified that mercury, selenium, chromium, manganese and cobalt were consistent with coal combustion, tracking economic activity in Asian regions, whereas arsenic, cadmium, nickel, antimony and zinc were more associated with non-coal combustion sources. The highest source of lead was liquid fuel combustion, including from traffic but a notable decrease in lead found in dust was seen after 2004, when lead was phased out of fuel mixtures. Krailertrattanachai et al. (2019) found heavy metal-contaminated roadside soils in rural areas of Thailand, when compared with soils further from the road. In their study, the concentrations of cobalt, chromium, copper, nickel, vanadium and zinc were found in levels toxic to plants, with cadmium, copper, lead and zinc concentrations showing a significant negative correlation with distance from the road. To better assess the health impact of particles in Bangkok, aerosols were sampled throughout 2018 and analysed to determine their metal content.

A monitoring campaign in northern Bangkok near to a toll road collected aerosol samples and measured aerosol concentrations throughout the year 2018 and on different size fractions. Size and metal analysis of these results can provide information on the characteristics of vehicular pollution in many heavily populated cities.

## Methods

### Study location

Between February and November 2018, particulate air pollution was measured in Lak Si, a northern district of Bangkok near a toll road (13.87°N, 100.58°E), covering three seasons (cool, hot and rainy). There are several classifications regarding seasons in Thailand but two broad seasonal categories exist, dry and wet and within the dry season there is a further distinction between hot and cool. The dry (cool) season runs from October through to February, and December is the coldest month with an average temperature of 25.6 °C, the average rainfall is 8 mm. The dry (hot) season runs from February to May, the average temperature in the hottest month, April, is 30.2 °C, the average rainfall is 67 mm. During the wet season from May to October the average temperature is between 29.7 °C in May and 27.9 °C in October, the month with the highest rainfall is September with 320 mm. All data from en.climate-data.org climate model based on data from the European Centre for Medium-Range Weather Forecast Data from 1999 to 2019, accessed on 17th January 2021. UV radiation levels are determined from tropospheric ultraviolet and visible (TUV) radiation model which vary between 5 and 10% throughout the year.

Within the grounds of the Chulabhorn Research Institute (CRI), two locations were chosen for long-term measurements, a rooftop location and an inlet from the 5th floor of a different nearby building. All instrument locations and dates of measurements are given in the supplementary materials (Table S1). The measurement site on the roof of the Chemistry Building was 26 m above ground and approximately 100 m away from the busy Don Muang toll road. The rooftop site measured PM_10_ and weather. An inlet into a 5th floor window 22 m above ground in a nearby building was used to measure particle number concentration (PNC) and size-separated samples of PM. PNC was also assessed using a hand-held condensation particle counter (CPC) by both spot measurements at three locations nearby, and the walking route between them.

**Fig. 1:**
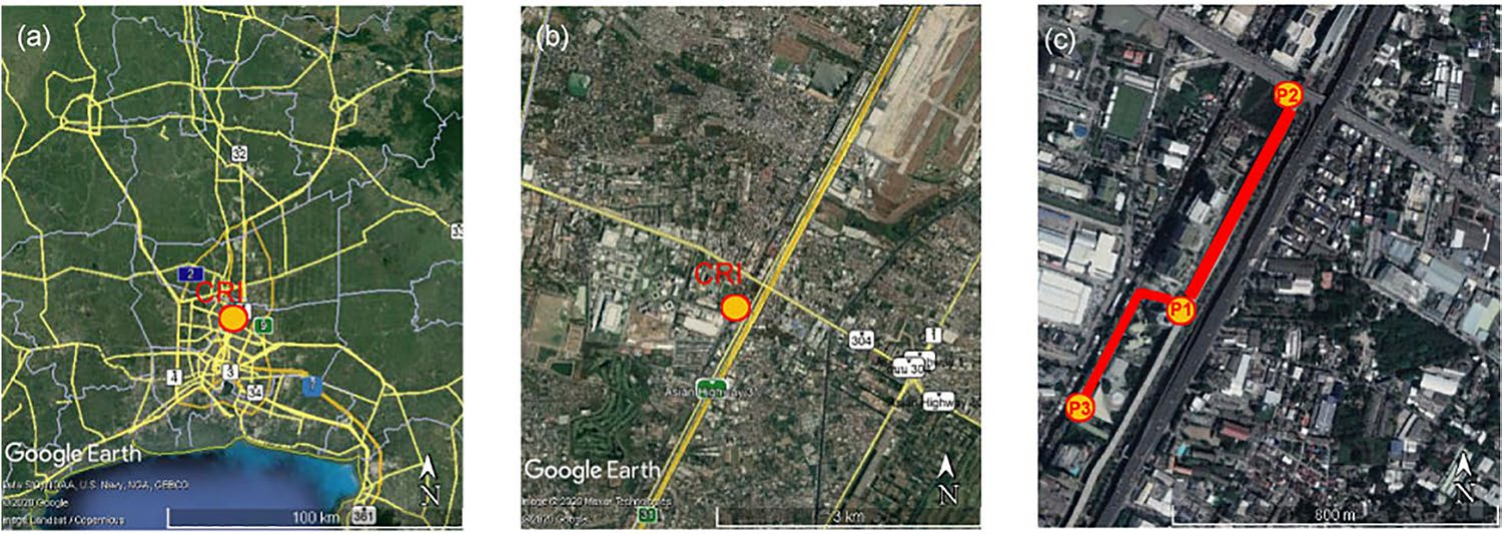
**a** Study location Lak Si in relation to the wider Bangkok area, **b** the study location within Lak Si and **c** the route in Lak Si taken when sampling aerosol using a hand-held CPC images from Google Earth

The measurement location is shown in Fig. 1 and is approximately 15 km to the north of Bangkok city centre. To the east, there is a state railway, and construction work is ongoing for the new elevated ‘red line’ railway, which continued throughout all measurements. To the west, across a small canal, is a built-up area comprising both residential and governmental properties, including building work for a new hospital building. The toll road leads NNE towards the Don Muang International Airport.

### PM_10_ mass sampling

PM_10_ samples were collected using a Sven Leckel LVS3 PM_10_ sampler which drew ambient air through a PM_10_ head at a flow rate of 2.3 m^3^ h^−1^. A total of 129 47-mm PTFE filters were weighed before and after sampling with a microbalance within an environmentally monitored weighing room. PM_10_ was collected for 24-h samples during weekdays (10 AM–10 AM, Monday to Thursday), and a 72-h sample over weekends (Friday 10 AM to Monday 10 AM). On some occasions, high mass deposition on the filter caused overloading, resulting in measurements finishing early. Total volume sampled was recorded by the instrument and used with the measured mass to calculate mass concentration.

### Particle number concentration

Particle number concentration (PNC) was measured using two condensation particle counters (CPC) and an electrical low pressure impactor (ELPI, Dekati). One CPC (Model 5.403, Grimm Aerosol Technik) was located within the CRI building on the 5th floor, facing towards the toll road (facing NNE) approximately the same height as the toll road, 100 m from the road. Samples were drawn through a 2 m long, conducting tube into the CPC. The building envelope will affect the CPC measurement, but by choosing a consistent location, temporal changes in PNC can be observed. Measurements continued through February, March and April, but could not continue through the rest of the campaign due to instrumental error. The ELPI measured from an inlet on the 5th floor of the CRI building for 3-day samples, covering weekdays or weekends. Filter masses were weighed and number concentration was measured by the ELPI through electrostatic measurements. Sizes were separated according to aerodynamic diameter.

To demonstrate how representative measurements from the 5th floor are to somebody walking roadside a portable CPC (Model 3007, TSI Instruments) was used on 15 occasions, carried at an adult waist height (~ 1 m) above ground level on a repeated walking route. This route incorporated three locations chosen for spot measurements and is illustrated in Fig. 1c. The first was at the ground floor in front of the CRI Biomedical Building entrance (e.g. at ground height near to the CPC measurements) (Fig. 1c, P1) after 3 min of sampling, the operator walked alongside the ground on the west side of Kamphaeng Phet 6 Rd, also west of the railway line, NNE towards the junction with Chaeng Wattana Road. The second measurement position was Lak Si Junction on the south side of Chaeng Wattana Road, opposite the IT Square shopping complex (P2). After a 3-min period had elapsed, the operator walked back along the same road SSW towards the CRI. Another 3-min spot measurement was made in front of the entrance to the Biomedical Building (P1). After the spot measurement, the operator walked behind the Biomedical Building, through a covered walkway, and continued to walk SSW to the east of the canal, but sheltered from the road by the complex buildings. At the rear of the CRI residence (P3) a final 3-min spot measurement was taken. CPC PNC was recorded at 1-s sampling intervals, and GPS position recorded on a mobile smartphone at 3 s intervals.

### Aerodynamic mass and number distributions

The ELPI is a cascade impactor which collects PM onto 12 filter stages, segregated by particle size. A total of 25-mm polycarbonate filters with 0.2-μm pore size were used for sample collection. The 1st stage, > 10 μm, uses a greased aluminium foil to ensure collection of the largest particles and to minimise particle bounce, but impaction grease could not be used on the other stages due to interference within the ICP-MS analysis.

The ELPI measures particle number size distribution in real time as each stage is connected to an electrometer, and particles are raised to a known charge distribution. The current in the electrometer can be used to calculate particle number size distribution and hence overall number concentration. The ELPI can be used to estimate mass concentrations from number concentration, but this facility was not used in this analysis. While the ELPI was set up to run with a final stage equipped with a filter to detect the remaining (< 20 nm) particles (the filter stage), an appropriate filter was not available for some measurements and so no particles were measured at the filter stage. The use of the filter stage requires the removal of the 12th stage, and the remaining stages had D50 cut off stages at 28, 93, 155, 262, 381, 612, 947, 1600, 2390, 6670 and 9900 nm. Before each measurement, the ELPI was tested for air leaks, and the electrometers in each filter stage were tested by flushing through with filtered air and setting the currents to zero.

### Weather

A Gill Instruments GMX501 measured temperature, pressure, humidity, solar radiation and wind speed and direction, while a Gill Instruments GMX100 measured rainfall using an optical method. Weather measurements started on 5 March 2018 and continued until 12 June when weather measurements went offline, measurements resumed on 6th August and continued until 3^rd^ October 2018, then resumed from 5th November until 15 November. Measurements were recorded at 1-s intervals, the wind speed and direction were used to find components along the WNW-ESE axis and the SSW-NNE axis which were chosen for analysis to represent winds parallel to and orthogonal to the major roads in the study location (see Fig. 1).

### Mass analysis

The LVS3 used 47-mm PTFE filters with 1-μm pore size to collect PM10 mass samples, and 25-mm nuclepore™ polycarbonate filters with 0.15- and 0.20-μm pore sizes used in the ELPI for size-separated sampling. The filters in the ELPI were chosen with smallest pore size available to minimise disruption of air flow that can be caused by using porous filters instead of aluminium foils.

Where possible, filter weighing procedure followed BS EN 12341:2014 standard method, although some deviations from standard procedure were necessary as relative humidity within the weighing room was often higher than the 50% limit designated in BS EN 12341:2014. Temperature and relative humidity were measured within the weighing room throughout the experimental campaign using an Omega data logger (OMYL-M90, Omega Engineering. Manchester, UK) so that temperature and humidity changes could be corrected in the analysis (see Supplementary Materials 2).

Both ELPI and LVS3 filters had the same weighing procedure, filters were weighed before and after the measurement being left in the weighing room at least for 1 day to condition to the ambient temperature and pressure. The filters were conditioned in partially covered Petri dishes, left ajar to allow air to circulate allowing volatiles to evaporate and the filters to be in a similar state before and after the measurement. The two filter blanks were measured before, during and after the sample filters were measured. Before the measurement, each blank was weighed six times to ensure consistency of the mass balance; subsequent to this, each filter was weighed four times, waiting for a stable measurement on the mass balance. Filters were placed in an ionised air flow before measurement to reduce electrostatic effects on weighing.

### Metal concentrations analysis by inductively coupled plasma-mass spectrometry

Ambient PM_10_ collected on filters was sonicated for 3 h at 69 °C in a sonication bath with 20 mL of 4% nitric acid. After sonication, the samples were filtered through Whatman 541 filter paper and diluted to 50 mL with deionized water. For sub-micron and ultrafine PM, air sample filters were digested in a mixture of high purity HNO_3_:HCl (4:1) by microwave digestion. The digested samples were subjected to metal analysis by inductively coupled plasma-mass spectrometry (ICP-MS) (Agilent 8900) which an internal standard solution containing Sc, Ge, Y, In, Rh and Bi in 1% HNO_3_ was simultaneously analysed. For quality control, reference material (NIST 1684a, urban particulate matter) was analysed with a recovery of 85–90%.

### Statistical analysis

Statistical tool used to interpret the composition of aerosols, include the enrichment factor (EF), hierarchical cluster analysis (HCA) and principal component analysis (PCA) (e.g. Ragosta et al. 2008; Pongpiachan and Iijima 2016). All calculations were performed in Python 3.7 (https://docs.python.org/3/, accessed January 2020) with Scikit-Learn (Pedregosa et al. 2011) and visualisations with Matplotlib (Hunter 2007) and Seaborn (Waskom et al. 2018).

### Health risk analysis

Health risk of exposure to trace metals bound to atmospheric particles was assessed according to methodology developed by the US Environmental Protection Agency (US-EPA). In this study, health risk assessments of metals were conducted for carcinogenic and non-carcinogenic risks from inhalation and ingestion (EPA US 1989; EPA US 2009a, b).

The exposure concentration for inhalation is proportional to concentration, exposure time and exposure frequency and inversely proportional to the average time. The average daily dose of carcinogen by ingestion is proportional to concentration, ingestion rate, exposure frequency and exposure duration and inversely proportional to body weight and average time. From these risks, hazard quotient and cancer risk were found (Liu et al. 2018; EPA US 2009a, b). Full details of the calculations can be found in Supplementary Materials 6.

## Results and discussion

### CPC particle number concentrations

**Fig. 2:**
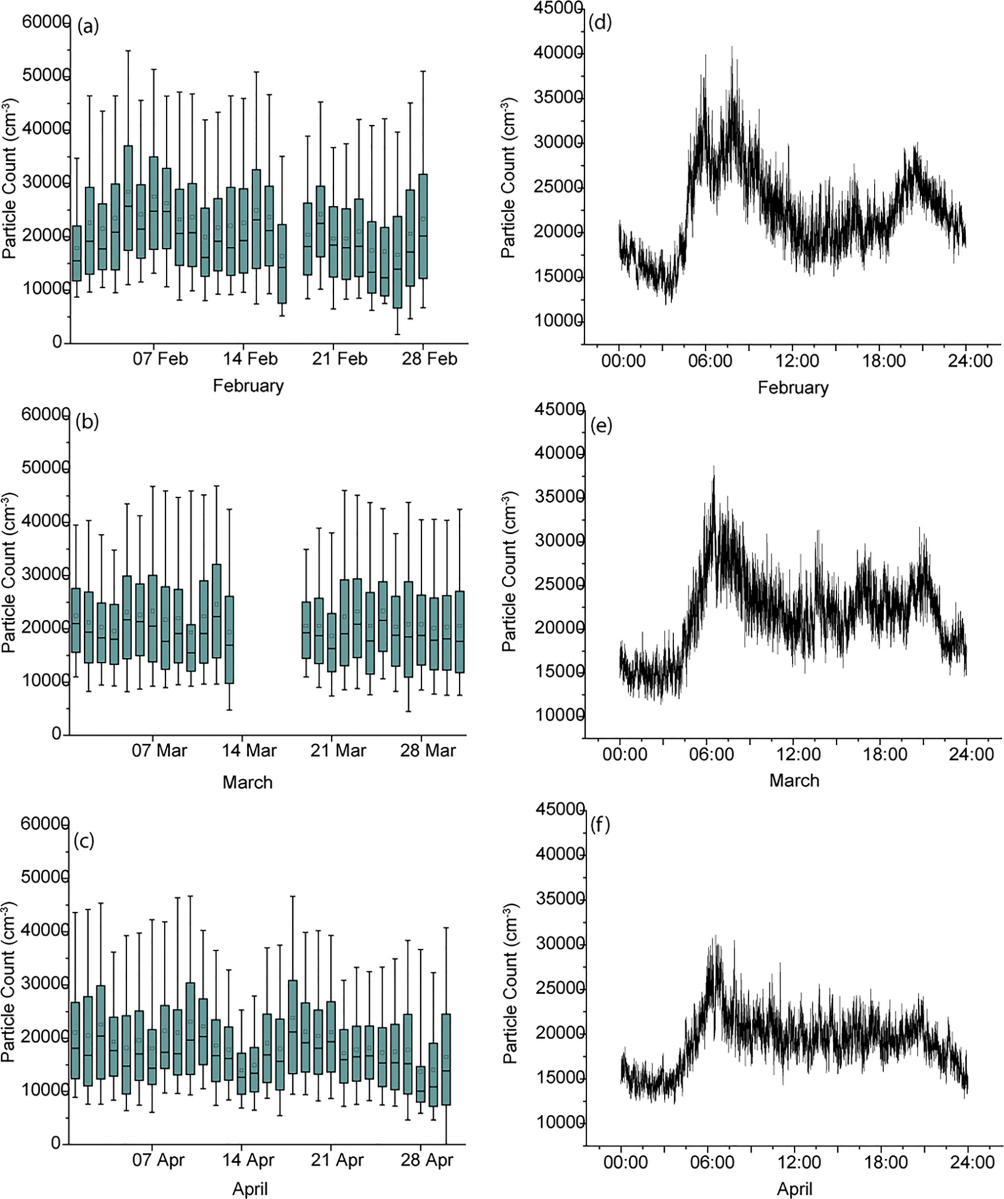
Concentration of particles measured at the CRI site over three months showing daily box plots for **a** February, **b** March and **c** April 2018 and the daily averaged time series for **d**, February, **e** March and **f** April

**Fig. 3:**
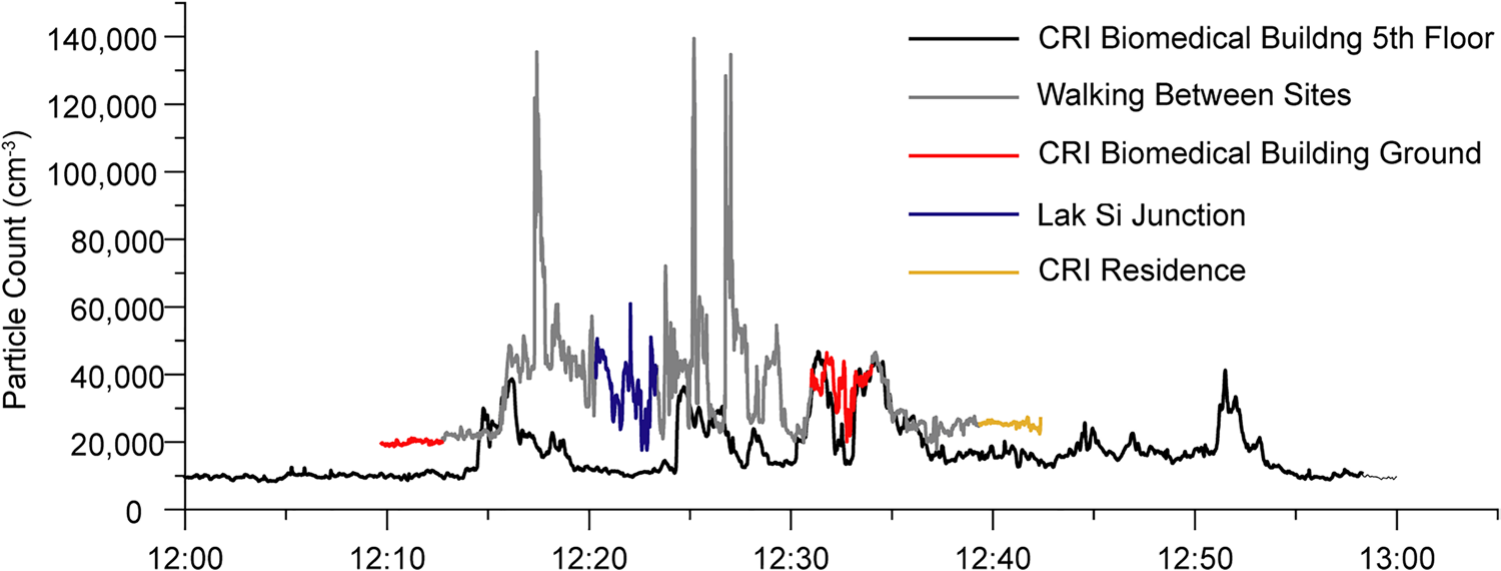
Time series showing the particle number count (PNC) measured from the 5th floor of the Biomedical Building (Grimm CPC), and measured by a hand-held particle counter (TSI CPC) on a walking route with 3-min spot measurements at 3 locations (outside Toxicology lab: red; near Lak Si Junction: blue; and behind the CRI residence: yellow) alongside a busy toll road in Lak Si, Bangkok, on 11 April 2018

The CPC on the 5th floor measured PNC in a fixed position throughout February, March and April 2018, with breaks for equipment maintenance until equipment failure stopped the measurement in May. The daily boxplots are shown in Fig. 2a, b and c for February, March and April, respectively. The daily average cycle was produced for each month by taking each second of the day and averaging them with the same second for every other day in the month. This allows the diurnal cycle to be seen, and for a monthly average PNC level to be calculated from the diurnal averages, which will avoid potentially missing data at peak times from distorting the average. Previous analysis of CPC and atmospheric potential gradient measurements at this location indicated a double diurnal cycle (Matthews et al. 2019) and the diurnal cycle in particle number concentration here shows a peak in the morning rush hour between 5 and 9 AM local time (UT + 7 h), and a broader peak in the afternoon rush hour in the February and March measurements, although in the April measurement daytime concentrations remain level until approximately 8 PM local time when concentrations drop in all three months. There is a significant reduction in particle count overnight. The mean (and standard deviation) of the 86,400 averaged seconds used to produce the monthly curves was found as this would mitigate against missing data points. These means are 22,124 (4631), 21,403 (4381) and 19,098 (3000) particles cm^−3^ for February, March and April, respectively. The TSI hand-held particle counter was used to measure PNC in three locations for 3-min durations, and also during the intervening walking route in order to test how representative the fixed 5th floor measurements were to a person walking in the street. Figure 3 shows an example time series of the walk taken on 11 April 2018 at noon (local time). The time series indicates 3-min spot measurements outside the CRI Biomedical Building (twice), near the Lak Si Junction centre and at the rear of the CRI residence. The time series from the 5th floor CPC is also shown, although due to instrumental differences in the range measured and response, data from both CPCs may not be directly comparable (see Supplementary Materials). The time series is typical of those recorded, with large peaks as vehicles passed near to the kerb in the walks between the Biomedical Building Ground floor and the Lak Si Junction. There were fewer peaks between the lab ground floor and the Residence as the measurements were shielded by buildings. The 5th floor CPC PNC also shows peaks, but these are lower in magnitude, as the additional distance between road and CPC inlet allows the aerosol to mix more with background air.

**Fig. 4:**
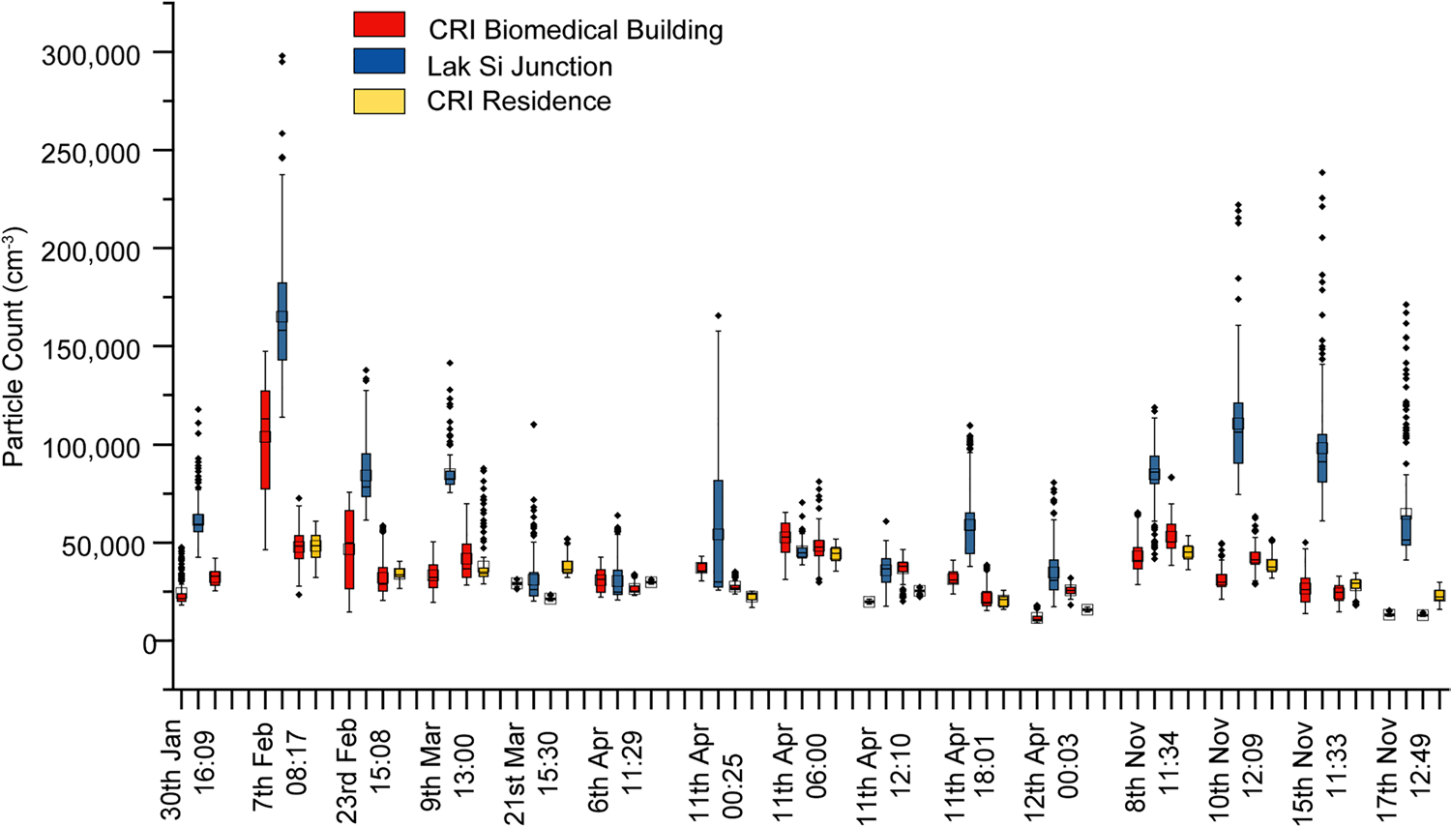
Box plot of three-minute samples of PNC measured in three locations during 15 survey measurements using a CPC carried at waist height. Measurements were taken at different times of day in different seasons

Figure 4 shows box and whisker plots of the 3-min spot measurements taken from each spot measurement location on the walking route. The measurements at roadside showed the highest average values and the largest spread of values (including outliers) compared with the two sites within the CRI compound as the measurement was closest to the source and emissions from vehicles are less well mixed. The roadside measurements showed the most variation, due to individual vehicles with high emissions passing at the time of measurement. As with the fixed CPC, the highest concentrations were found when measurements were taken in the morning. Five measurements were taken on the same day, 11 April 2018 starting at midnight, with a measurement every 6 h until midnight the following day. There was a very high signal at Lak Si Junction in the first midnight measurement, but excepting that, the results follow the pattern shown in Fig. 2d showing the daily average of measurements over all of April 2018 from the 5th floor. There is a rush hour peak at ~ 8 AM, and the overnight measurements are low, with the noon and 6 PM measurements at a similar level.

### Mass, number and metal distributions

**Fig. 5:**
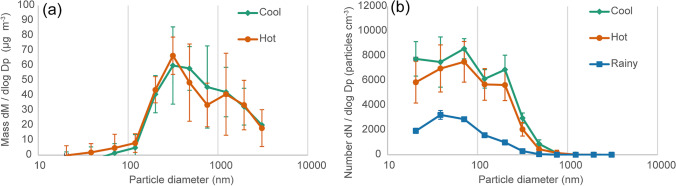
Seasonal averages of **a** mass size distribution measured on each stage of the ELPI for and **b** number size distribution measured on each stage of the ELPI for 3-day sample. Data sets show the average and standard deviation of each season, there were six measurements in hot season, four measurements in cool season and three measurements in rainy season. Mass (*M*) and number (*N*) concentrations have been normalised by dividing by the bin width size of each impactor stage (Dp) on a log scale as is commonly used to represent typical size distributions encountered in atmospheric aerosol science. The mass distributions were not available for the rainy season

Mass and number size distributions for all ELPI measurements are summarized in Fig. 5a and b showing normalised log-normal distributions (Heintzenberg, 1994) more details can be found in supplementary materials 3. Both dry seasons (hot and cool) show similar distributions in both number and mass, with the cool season showing a slight increase over the hot season (Fig. 5), and a peak in mass near to 400 nm. This peak is lower than that measured by Dejchanchaiwong et al. (2020) who showed a bimodal distribution with one peak at measured at the Kasetsart University in the 2018–2019 cool season; a site 5 km south of our measurement position around 500 m from the major roads. The smaller sizes measured in our site may be due to vehicular emissions being less aged. The rainy season sees a reduction in particle number, particularly at the lower sizes. Distributions were similar in both weekend and weekday measurements (see Supplementary Fig. S3).

**Fig. 6:**
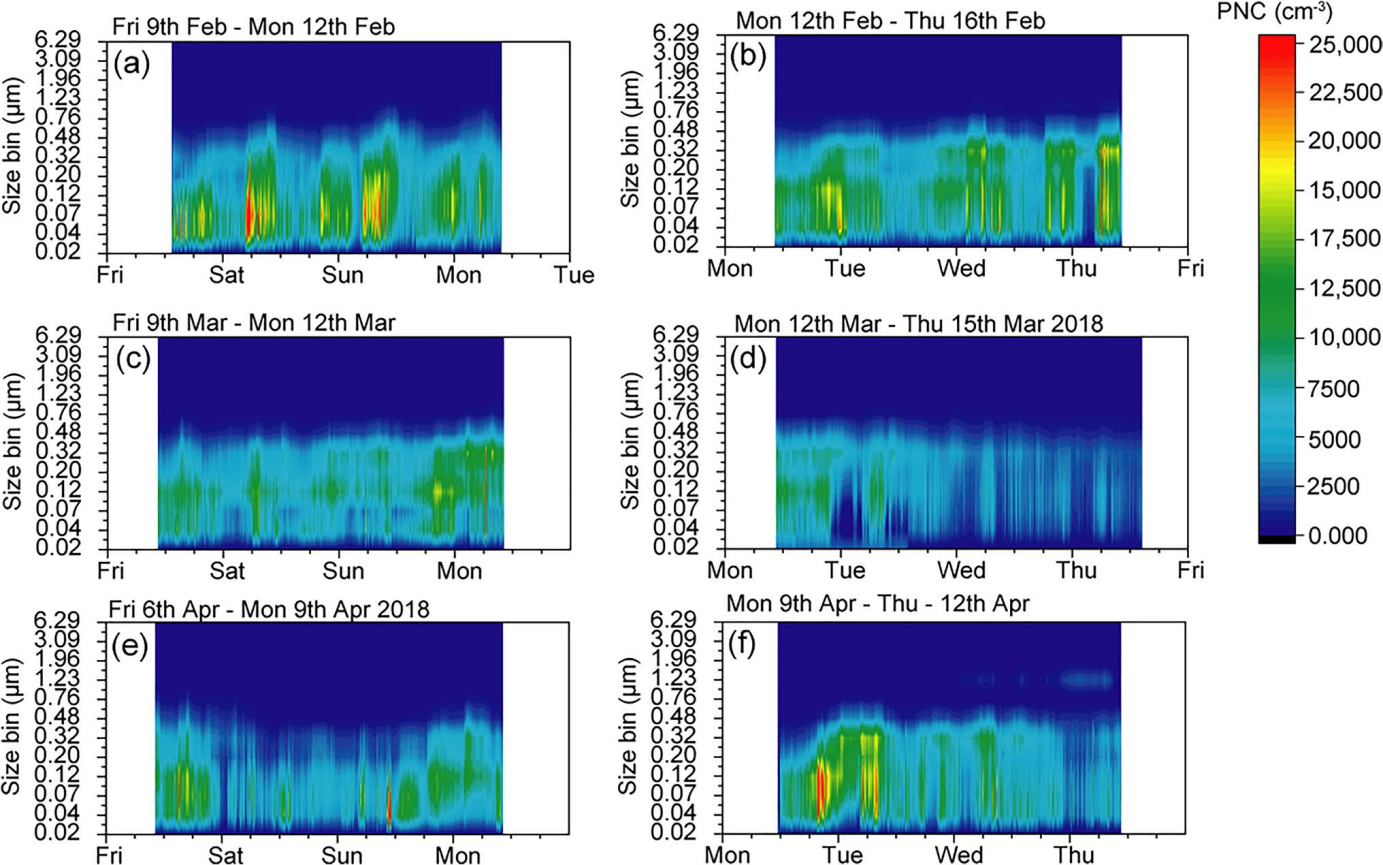
Example colour plots of the ELPI size distribution measured for three days in February, March and April. Weekend measurements are to the left and weekday measurements to the right

Selected colour plots (Fig. 6) show the PNC for the 3 days within the measurement. As shown in Fig. 5, the largest number concentrations are in the sub-micron range, but Fig. 6 indicates that the highest temporal variability is shown in the sub 100-nm (ultrafine) range.

**Fig. 7:**
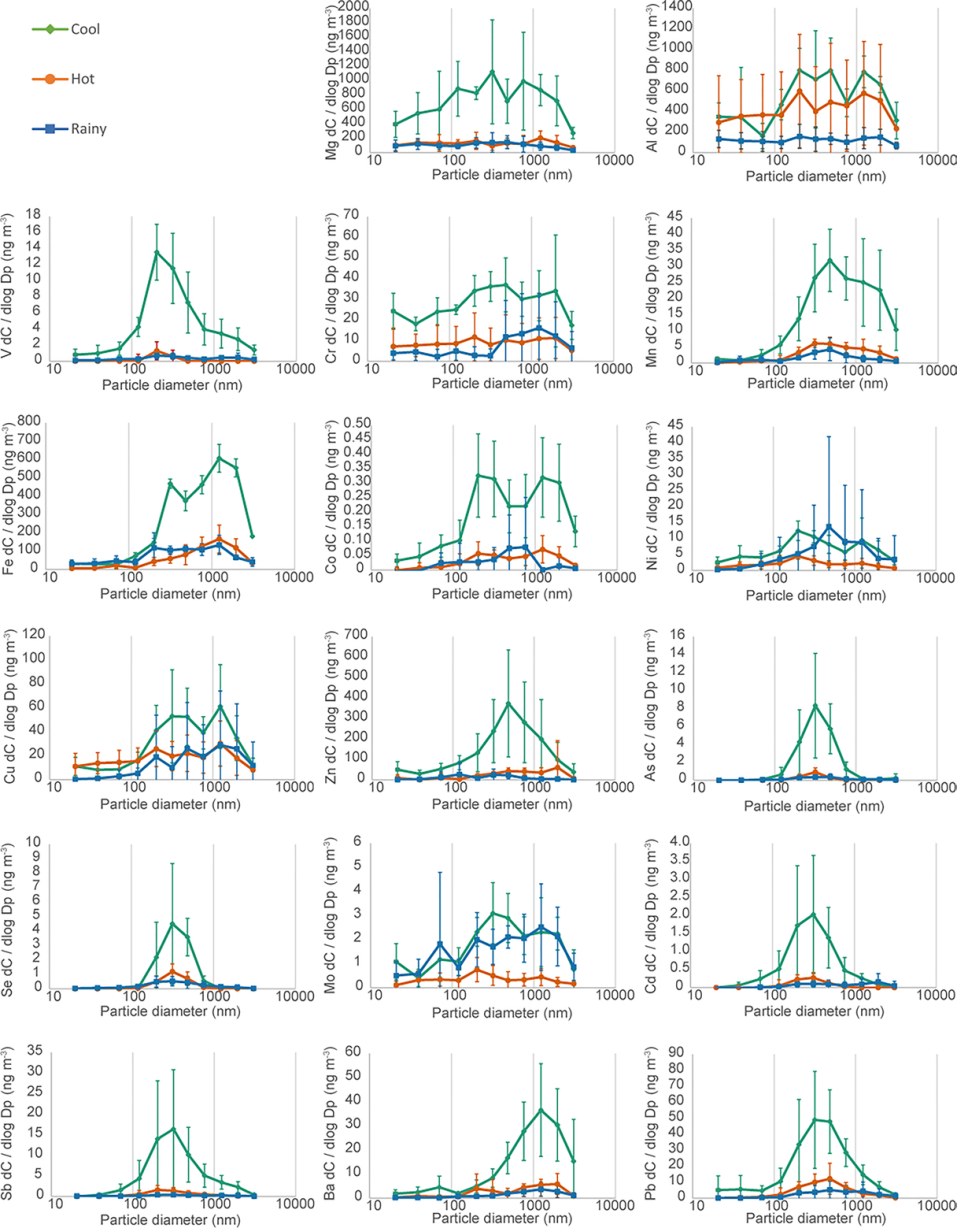
Airborne concentration of metal and metalloid within size-separated sampled of PM measured in fifteen sets of 3-day samples near a toll road in Bangkok. Data sets show the average and standard deviation of each season, there were six measurements in hot season, four measurements in cool season and five measurements in rainy season. Metal concentrations (*C*) have been normalised by dividing by the bin width size of each impactor stage (Dp) on a log scale, as is commonly used to represent typical size distributions encountered in atmospheric aerosol science. A version of this figure without normalisation can be found in supplementary materials

Metal and metalloid concentrations found on aerosol per unit of air sampled using ICP-MS for each filter stage measured by the ELPI are shown in Fig. 7, and in more detail in Supplementary Materials Section 3. Distributions vary in each element indicating different sources. Concentrations are highest in the cool season (Feb 2018) and are reduced in the hot season (Mar–Apr 2018). Metals that are highest in the largest size fractions (cobalt, copper, iron, barium) may come from non-exhaust traffic sources such as brake and tyre wear (Gietl et al. 2010; Harrison et al. 2012), whereas elements largely present in the sub-micron range (arsenic, selenium, cadmium, antimony) may have combustion sources, which would be predominantly traffic exhaust (Pulles et al. 2012). The metals with the highest concentrations in the sub-micron range were magnesium, aluminium, iron, zinc, copper and chromium. Phairuang et al. (2021) also showed that iron, copper, magnesium, zinc, chromium, potassium, sodium and lead were high in the ultrafine (PM_0.1_) range and Kanjanasiranont et al. (2022) found that copper, chromium and iron were dominant in the ambient PM_10_ concentration in Bangkok and its vicinity areas including Nonthaburi and Nakhon Pathom, Thailand.

### Seasonal PM_10_ measurements

**Fig. 8:**
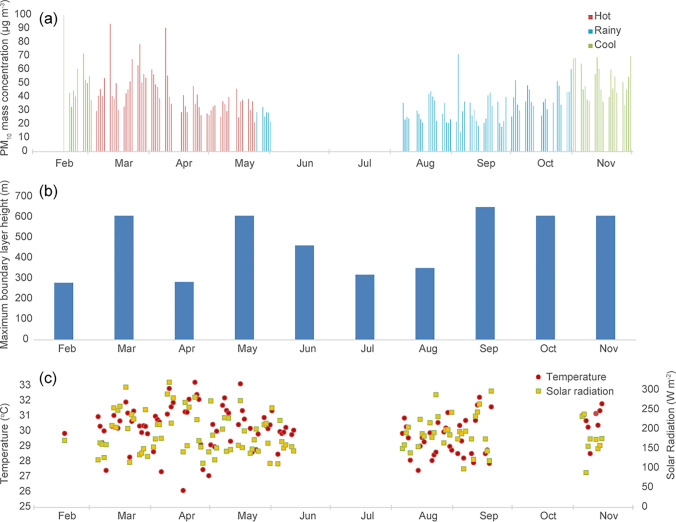
**a** Mass concentration (μg m^−3^) of PM_10_ samples taken in Bangkok between February and November 2018, covering 4 seasons in total (cool, hot, rainy and then cool again), **b** calculated maximum boundary height for the month and **c** mean average daily temperature and solar radiation from the Lak Si site where measurements exist showing highest temperatures in March and April

PM_10_ mass measurements over three seasons (cool, hot, rainy) are shown in Fig. 8a, where each measurement represents 24 h (weekday) or up to 72 h (weekend). The figure also shows the monthly maximum boundary layer depth for Bangkok, calculated using a 3-D dimensional chemistry and transport model, STOCHEM-CRI, more details about the boundary layer treatment can be found in Khan et al. (2021). In addition, the figure shows the daily temperature and solar radiation, measured on the CRI rooftop. The highest temperatures were shown in March and April, during the hot season.

The measurements show a decrease in PM_10_ from cool season into hot season, and the lowest masses were measured in the rainy season. Mass measured increases when the rainy season ends at the beginning of November. The cause of the seasonal difference could be due to the lack of rainfall causing washout within the dry (cool and hot) seasons compared with the rainy season. The lower boundary layer during the cooler months may also cause a trapping of aerosol in the urban canopy and therefore increasing concentrations (Pongpiachan and Iijima 2016; Watcharavitoon et al. 2013; Dejchanchaiwong et al. 2020). Calculated maximum boundary layer heights in Bangkok do not show a clear seasonal trend, and do not support this suggestion, but we did not run measurements for most of December and January, which are often the cooler months, and multiple years of measurements are required to identify seasonal trends.

A further source of aerosol in the dry months in South East Asia is rural crop burning (Sahu et al. 2011; Aman et al. 2020; Dejchanchaiwong et al. 2020), although traffic aerosol is a larger contributor. Biomass burning peaks occur in Thailand and surrounding countries between December and March (Vadrevu et al. 2019), and Dejchanchaiwong et al. (2020) identified that high PM_2.5_ and PM_0.1_ concentration haze events in Bangkok during December 2018 were likely to be caused by aerosol transport from Cambodia and central and northeastern Thailand. Transmission electron microscopy (TEM) analysis of PM_1_ particles measured between January and February 2020 in Bangkok revealed chains of spherical particles typical of combustion sources (Kanjanasiranont et al. 2022). We measured some of the highest masses in February, March and April. A trajectory analysis was carried out using the Hybrid Single-Particle Lagrangian Integrated Trajectory Model (HYSPLIT) of the National Oceanic and Atmospheric Administration (NOAA) (Stein et al. 2015); the details of the analysis are shown in Supplementary Materials 4. On selected dates, air masses from Cambodia, Vietnam and Laos, to the East of Bangkok, may reach the city carrying biomass burning aerosol, although concentrations may be small compared with traffic emissions (Dejchanchaiwong et al. 2020).

Mass concentrations of selected metals and metalloids from whole PM samples were measured by ICP-MS and the average concentrations per season are shown in Table 1. The enrichment factor is used to describe the amount of an element that is higher than the proportion within the Earth’s crust, as shown in Eq. 1 for element *E* compared with the reference *R*.

**Table 1:** Average mass concentration (μg m^−3^) and metal concentrations (ng m^−3^) for PM_10_ measurements taken between February 2018 and November 2018 split into 3 seasons (hot, rainy and cool)

Season	Mass	Mg	Al	Ca	V	Cr	Mn	Fe	Co	Ni	Cu	Zn	As	Se	Mo	Cd	Sb	Ba	Pb
Hot	42.65	1984.76	1291.95	13163.13	36.12	19.96	126.53	3601.34	1.92	20.55	180.83	980.87	16.35	12.50	3.96	2.92	29.38	208.20	114.08
Rainy	42.45	1132.46	1195.64	7157.38	19.61	15.22	116.07	2580.48	1.55	14.79	129.83	1010.06	17.00	11.35	3.27	3.02	25.98	141.42	130.42
Cool	51.70	1341.83	1288.29	18309.18	28.19	19.97	197.53	2970.30	2.34	12.15	183.91	1226.41	24.53	20.13	3.32	7.54	51.18	211.19	211.60
All	44.87	1464.25	1223.48	10870.26	26.92	17.80	129.57	3008.01	1.78	16.68	157.45	1046.01	17.80	13.00	3.55	3.57	30.60	175.11	133.94

1 $$\mathrm{EF}= \frac{{\left(E/R\right)}_{Air}}{{\left(E/R\right)}_{Crust}}$$

**Fig. 9:**
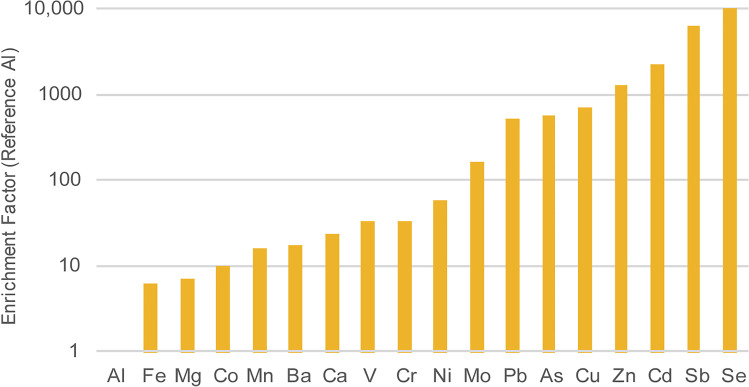
The enrichment factor of elements measured in PM_10_ air samples in Bangkok compared to crustal samples found in Wedepohl (1995)

Choosing an element that is predominant in the Earth’s crust (typically iron, aluminium or silicon) and using upper crust data from Wedepohl (1995), the ratio of elements in the air (as measured in PM_10_) over the ratio of elements in the crust gives an indication of which elements have the highest non-crustal sources. Aluminium was chosen as the reference, as it had the lowest air/crust ratio, and the results are plotted in Fig. 9. Elements that have a very high non-crustal source include copper, zinc, arsenic, selenium, molybdenum, cadmium, antimony and lead which all have an enrichment factor > 100. The highest concentration in PM_10_ measurements was the crustal source metals of calcium, magnesium and iron.

In order to identify the sources of PM_10_, we followed the method in Pongpiachan and Iijima (2016) and used PCA which group metal concentration signals such that each successive orthogonal component explains the maximum amount of total variance in the data. While metal concentrations and meteorological data were not normally distributed (Kolmogorov-Smirnov 2-tailed test), the conclusions from HCA and PCA can still be considered valid as we do not undertake hypothesis testing.

**Fig. 10:**
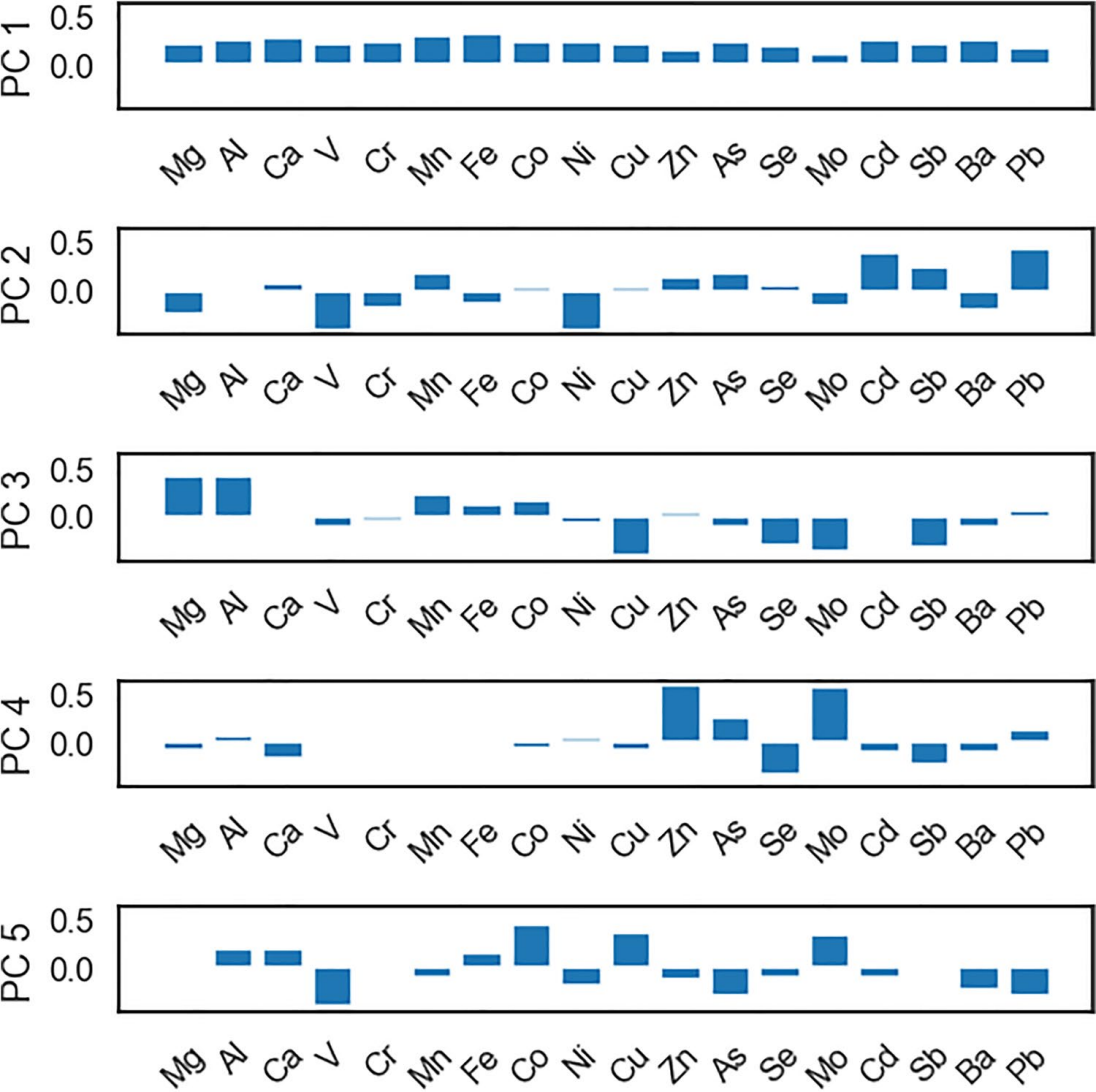
Principal component analysis of PM_10_ elemental concentrations

**Fig. 11:**
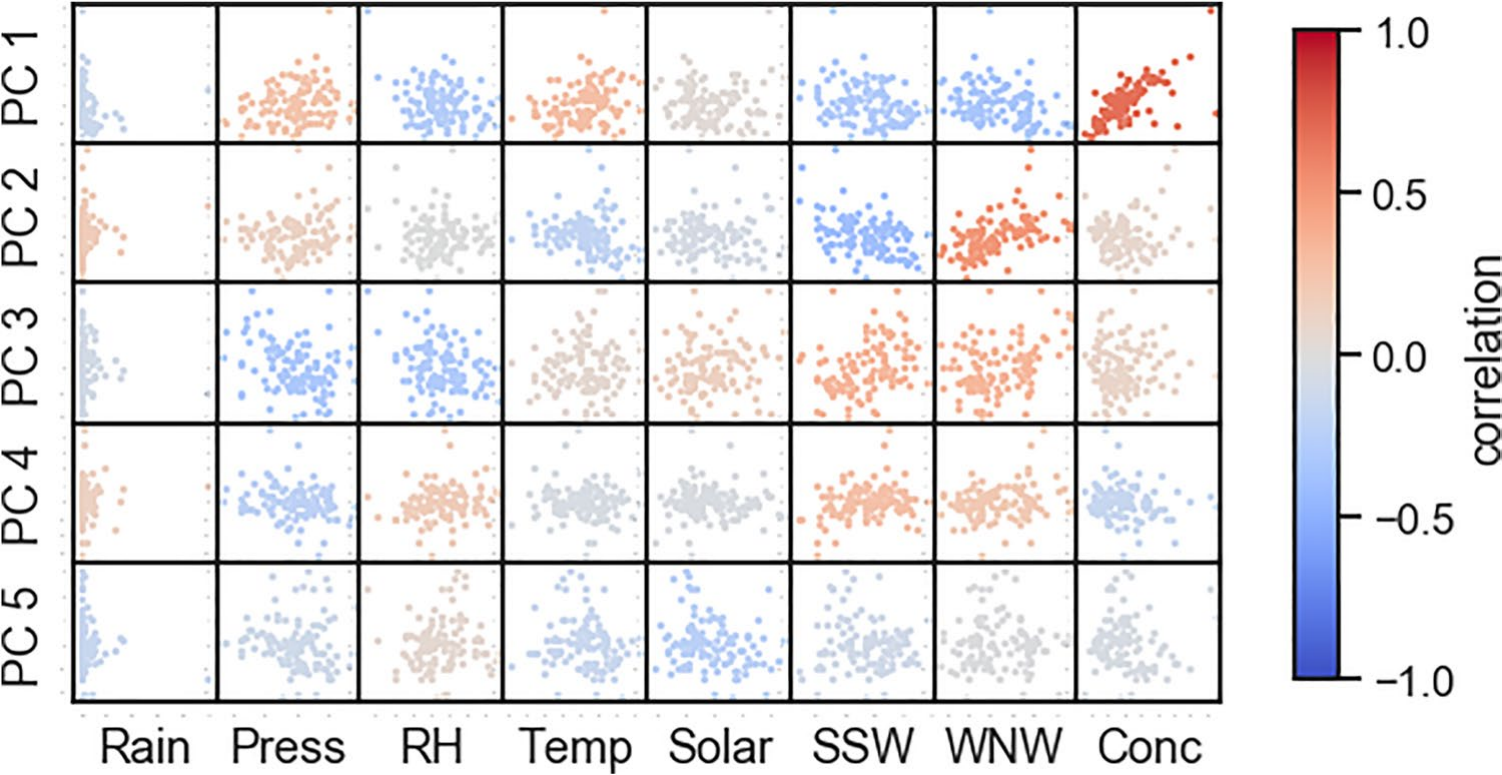
Correlation matrix showing the first five principal components against measured weather conditions and mass concentration during the measurement period

The metal signals were first centred and scaled by their means and standard deviations and then PCA performed; the first 5 principal components (PC1 to PC5) are shown in Fig. 10 (the explained variance for each principal component is discussed in Supplementary Materials 5). There is no step change in the amount of variance explained by each component, beyond the first, and so we chose to keep the first five components based on the number of probable sources. These five components explained 78% of the variance. To potentially assign these components to sources, we plotted each against the total concentration, rainfall, pressure, relative humidity, temperatures, solar insolation and the wind speed in the SSW and WNW directions (to align parallel with, or orthogonal to the major roadways) (Fig. 11).

Within the principal component analysis, PC1 is related to the total mass of the samples, and this explanation is supported by the strong correlation between PC1 and the total mass measured in Fig. 11. PC2 clusters together manganese, cadmium, antimony, arsenic and lead, most of which are metals associated with tailpipe emissions (Pulles et al. 2012; Pongpiachan and Iijima 2016) and these are high while elements related to non-combustion emissions (vanadium, nickel, barium) are low; PC2 can therefore be considered the result of combustion aerosol. Comparing with the metal size distributions in Fig. 7, we see that elements associated with combustion are concentrated in the sub-micron range, and the non-combustion vehicle emissions are found mainly within aerosol greater than 1 micron. Therefore, it is possible that a positive association with PC2 represents the smaller particles. The correlation with WNW winds implies that PC2 is strongest with WNW or NNE winds (as PC2 is inversely related with SSW winds) which may be surprising as it would be expected that combustion aerosol would be strongest with wind directions drawing aerosol from the major roadways, as the smaller aerosols are shorter lived (Kittelson 1998). One explanation may be that the level of tailpipe emissions is seasonal; the predominant wind direction changes through the year. Westerly winds are predominant in the months of June to September, while Southerly winds are predominant during October. Measurements of metal concentrations in this size range in Bangkok show that arsenic is elevated in the hot season (March to June) at a roadside site when WNW winds are more prevalent (Matthews et al. 2020), which implies that these increases are seasonal. Another explanation may be that local dynamic forces are preventing the smaller particles produced at the road from reaching the measurement position.

The relationships between the PCs and meteorological conditions weakens after PC3. PC4 may show a slight association with wind directions SSW and WNW and may relate to increased pressure and low relative humidity, but the relationship is weak while PC5 shows no correlation with wind direction. PC4 and PC5 only account for 5% or less of overall variance (see Supplementary Materials, Fig. S11).

**Fig. 12:**
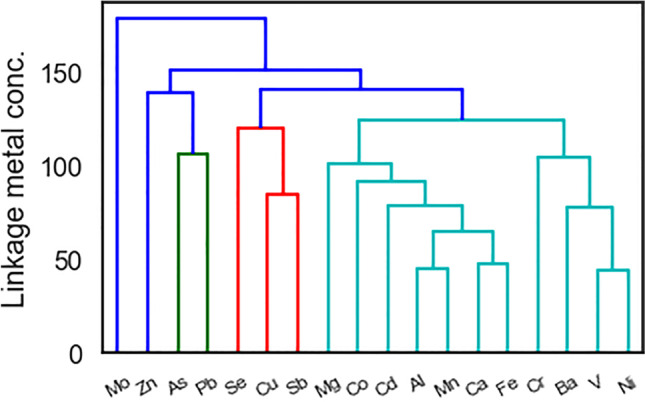
Dendogram showing the relationships between 18 elements for the measured PM_10_ concentrations

As an alternative to PCA, we performed HCA to see the correlation structure of the metal signals. The same standardised variables were used as in the PCA, except that this data matrix was transposed so that each time point was a ‘variable’. Using SciPy (Virtanen et al. 2019), HCA was performed with the ‘average’ linkage function and the squared Euclidean distance metric. The resulting dendrogram measures the correlation between metal signals (see Fig. 12).

Pongpiachan and Iijima (2016) identified three main sources for PM_10_ in Bangkok from its metal content: crustal emissions, vehicle exhaust and vehicle brakes and tyres. The strong cluster of aluminium, manganese, calcium and iron in Fig. 12 is consistent with Pongpiachan and Iijima who identified aluminium, manganese and iron as from crustal sources. The second largest cluster from Pongpiachan and Iijima was exhaust which is often marked by lead, vanadium, cobalt and zinc. In our analysis, zinc, arsenic and lead are clustered together, as are selenium, copper and antimony. Pulles et al. (2012) identified these as from tailpipes, either in the fuel mix or the lubricant mix. The former may be due to common traffic sources. Non-combustion emissions were identified by Pongpiachan and Iijima through the inclusion of barium, and linkage between chromium, barium, vanadium and nickel may be related to non-traffic emissions.

### Health risk assessment

A health risk assessment, carried out according to the methods of US-EPA (details in Supplementary Materials 6), was used to assess the inhaled and ingested health risk according to these measurements. Risk from airborne pollutants can occur from either inhaled or ingested (food, drink and hand-to-mouth) routes. The non-carcinogenic risk by inhalation is above the safe limit for lead, manganese and arsenic. For ingested particles, antimony and arsenic were the highest risk, but below the safe limit. In the size fractionated data, the risk was highest in the range of 0.11–1, where a large concentration of metals can be found (Fig. 7).

The carcinogenic risk by ingestion for metals bound to PM_10_ is highest for nickel, arsenic, cadmium and lead, but is overall small. In the fine and ultrafine range, there is an increased risk of ingestions in nickel and chromium. Considering the toxicity, many of the carcinogens of concern (arsenic, cadmium, chromium, nickel, lead, antimony) are found at more than ten times the crustal ratio. Chromium and nickel are found across all size ranges, while cadmium, arsenic and lead are predominantly in the sub-micron range. This may affect health outcomes of exposure to these particles, due to smaller particles exhibiting a higher specific surface area and potential deeper penetration within the lung. However, health impact analyses show ingestion may be more impactful. Our ICP-MS analysis did not provide information on the valence state of metals, which may impact on the health implications for inhalation of aerosols containing these elements, where particular valences exhibit more toxicity e.g. Cr(VI) compared with Cr(III). Therefore, ion-chromatographic (or other suitable) methods may provide additional information relevant to health outcomes for these elements.

## Conclusions

PM was sampled in one location in Bangkok and analysed for metal content, for short times PM was measured over different size fractions. Particle number concentrations were measured in the local area. PM_10_ is elevated during dry seasons, particularly the cool season. The ELPI measurements tended to have the lowest number and mass concentrations in the hot season (March–April). Using known chemical tracer species, aerosol measured in Lak Si, Bangkok, contains crustal sources (indicated by iron, calcium and aluminium), combustion sources (indicated by arsenic and lead) and tyre (vanadium) and brake dust (barium). The data here indicate that metals associated with traffic combustion sources are more likely to be found on the smaller particles (sub-micron), and non-combustion sources on larger sources (PM_2.5_). By analysing the differences in carbon structure in PM_2.5_ and PM_1_ in aerosols in urban Bangkok, Vejpongsa et al. (2017) conclude that PM_1_ is created almost exclusively from combustion sources. Very few measurements of size fractions lower that PM_2.5_ have been measured in Bangkok, but Phairuang et al. (2021) did make a thorough study of metal in small size fractions in Bangkok and found that PM_0.1_ is slightly higher in the cool season than at other times of the year, and declined from November to July in agreement with our ELPI and CPC measurements. A PCA analysis found diesel exhaust to be the largest source of metals in both dry seasons, while industrial processes were highest in the rainy season.

Particle number concentration had a clear diurnal cycle in the CPC measurements from the 5th floor of the Biomedical Building, with peaks during the morning and evening rush hours. Aerodynamic size distribution also had greater temporal variability in the particle sizes below 300 nm, as shown by the ELPI measurements, although individual days did not show a consistent pattern. Survey measurements taken by a hand-held CPC showed great variability in the particle number concentration, especially when roadside and during heavy traffic times during the day. These measurements combined reinforce the heterogenous nature of the smaller particles.

The measurements here show that the sub-micron range can be different chemically and may be more heterogenous than larger particles. The exposure of populations to the smaller particles, therefore, is highly dependent on both where and when they are exposed. A population that spends more time roadside will therefore receive a higher dose than an urban background measurement may suggest. It may be that more frequent measurements of these smaller particles will be required alongside PM_10_ and PM_2.5_ data sets in multiple urban locations to properly evaluate the health effects of urban aerosol.

## Data

Citable as:  Matthews, J. (2022): PM10 concentration and composition measurements in Lak Si, Bangkok, Thailand. Centre for Environmental Data Analysis. http://catalogue.ceda.ac.uk/uuid/43a22be3b71d46f0a5d02c7ee3472d24

## Supplementary Information

Below is the link to the electronic supplementary material.

Supplementary file1 (DOCX 5.64 MB)

## Data Availability

Data will be available after publication through the CEDA database.
