# Supplementary material for: Aerosol mass and size-resolved metal content in urban Bangkok, Thailand
Source: Environ Sci Pollut Res Int. 2022 Jun 15;29(52):79025–40. doi: 10.1007/s11356-022-20806-w (PMC9587116; doi:10.1007/s11356-022-20806-w)
Supplement: Supplementary file 1 — Supplementary file1 (DOCX 5.64 MB) [file 11356_2022_20806_MOESM1_ESM.docx]

Aerosol Mass and Size-resolved Metal Content in Urban Bangkok, Thailand

Supplementary Materials

James C Matthews^1^^[[1]](#footnote-1)^*, Panida Navasumrit^2^, Matthew D Wright^1^, Krittinee Chaisatra^2^, Chalida Chompoobut^2^, Robert Arbon^1,3^, M Anwar H Khan^1^, Mathuros Ruchirawat^2^ and Dudley E Shallcross^1,4^

*^1^ School of Chemistry, University of Bristol, Cantock’s Close, Bristol, BS8 1TS, UK*

*^2^* *Laboratory of Environmental Toxicology, Chulabhorn Research Institute, Bangkok 10210, Thailand*

*^3^ Jean Golding Institute, Royal Fort House, University of Bristol, Bristol, BS8 1UH*

*^4^ Department of Chemistry, University of the Western Cape, Robert Sobukwe Road, Bellville, 7375, South Africa*

**Supplementary Materials 1: Measurement table**

Within this study three instruments were used for measuring aerosol particle number concentration (PNC). These each have different measurement ranges and techniques so direct comparison between instruments is problematic, but, some cross comparisons were made throughout the project which indicate what comparisons can be made.

**Table S1.** Instruments and measurement periods during study period.

| **Instrument** | **Manufacturer** | **Parameter** | **Sample time** | **Time span (all 2018)** | **Notes** |
| --- | --- | --- | --- | --- | --- |
| LVS3 | Sven Leckel | PM10 mass  PM10 metal content | 24 hours (WD)  72 hours (WE) | 16^th^ Feb to 12^th^ June  6^th^ Aug to 30^th^ Nov |  |
| ELPI | Dekati | Mass distribution  Number concentration  Metal content | 72 hours (mass)  1 s (number) | 12^th^ – 15^th^ Feb  16^th^ – 17^th^ Feb  23^rd^ – 26^th^ Feb  26^th^ Feb – 1^st^ Mar  9^th^ – 12^th^ Mar  12^th^ – 15^th^ Mar  23^rd^ – 26^th^ Mar  26^th^ – 29^th^ Mar  6^th^ – 9^th^ Apr  9^th^ – 12^th^ Apr  4 – 7th^th^ May  7^th^ – 10^th^ May  1^st^ – 4^th^ June  4^th^ – 7^th^ June  8^th^ – 11^th^ June | No mass or PNC  No mass or PNC  No mass  No mass  No mass |
| PC | Grimm | Particle count | 1 s averaged to 1 minute | 1^st^ Feb to  17^th^ Feb  19^th^ Feb to Mar 13^th^  19^th^ Mar to Apr 30^th^ | Measurements stopped in May due to instrument malfunction |
| CPC | TSI | Particle count | 1 s | 30^th^ Jan PM  7^th^ Feb AM  23^rd^ Feb PM  9^th^ Mar PM  21^st^ Mar PM  6^th^ Apr Noon  11^th^ April night  11^th^ Apr AM  11^th^ Apr Noon  11^th^ Apr PM  12^th^ Apr Night  8^th^ Nov Noon  10^th^ Nov Noon  15th Nov Noon  17^th^ Nov Noon |  |
| GMX501  GMX100 | Maximet | Weather: Temperature, pressure, relative humidity, wind speed, wind direction  Rainfall | 1 s | 5^th^ Mar – 12^th^ Jun  6^th^ Aug – 3^rd^ Oct  5^th^ Nov – 15^th^ Nov | Data logger failure  Data logger failure |

**Table S2.** Dekati Electrical Low Pressure Impactor stage cut off (nm)

|  | Filter stage | 1 | 2 | 3 | 4 | 5 | 6 | 7 | 8 | 9 | 10 | 11 | 13 |
| --- | --- | --- | --- | --- | --- | --- | --- | --- | --- | --- | --- | --- | --- |
| D50 |  | 28 | 93 | 155 | 262 | 381 | 612 | 947 | 1600 | 2390 | 6670 | 9900 |  |
| Geometric mean | 10 | 21 | 39 | 71 | 120 | 202 | 316 | 483 | 761 | 1231 | 1956 | 3088 | 6285 |

**Supplementary Materials 2: Temperature and Humidity Correction**

Due to large changes in temperature and humidity within the weighing room, a correction was made to the mass of the filters used within the ELPI, 25 mm diameter nucleopore filters. Throughout the weighing process, two filter blanks were kept within the weighing room, B1 and B2. The mass of both filters were assessed (six repeats) before any sample filters were weighed, four times during the middle of the measurement, and four times after the measurements had finished. Temperature (T) and relative humidity (RH) were recorded every five seconds during the measurement period using an Omega OMYL-M90 data logger. In total there were 42 measurements (14 sets of three) for each of B1 and B2.

A multiple regression was run using R with the measurements from the measured masses of B1 (M_B1_), and the average T and RH measured during the weighing period. The relationship between the M_B1_ and the filters original mass m_B1_ with respect to T and RH can be described by Eq. S1:

M­_B1_ = m_B1_ + C1.T + C2.RH (S1)

where C1 and C2 are constants. Multiple regression found the parameters C1 = -0.03928 µg °C^-1^ and C2 = 0.025071 µg when T and RH were measured in g and %, with the mass of filter B1 at T = 0 C and RH = 0%) found to be 31.1863 µg.

To verify this relationship, the mass measured from the filter blank, M, was plotted against the mass predicted from m for each of the 42 mass measurements. Figure S1 shows the mass measured, M (y axis) against the mass calculated from m, C1, C2 and the measured temperature and humidity (x axis) for all measurements of B1, indicating a very good match between predicted and measured, with a trend line plotted through the origin. The correlation coefficient for the measured versus predicted mass was 0.944, showing a strong correlation.


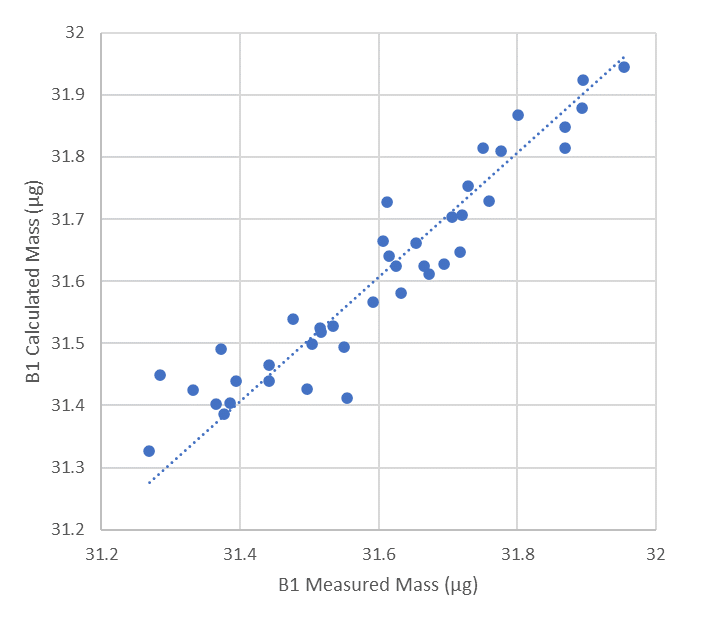


**Figure S1:** Calculated mass from temperature and humidity measurements in the weighing room against measured mass for filter blank B1.

To further test the validity of this correction, the corrected mass of B2 was calculated from the same constants, C1 and C2. Using the first measurement (MB2,1) C1 and C2 were used to find a value for m_B2_ = 30.52653 µg. That m_B2_ was then used in equation 1 to calculate the predicted temperature and humidity corrected masses for B2. Figure S2 shows the measured and predicted masses of B2, with a regression line fit set to intercept at zero. The correlation coefficient for this relationship was 0.959.

Having established that the relationship between m, T and RH can reliably predict the masses of the filter blanks, this formula has been used to correct the values of measured filter masses before and after sample collection. Using measured temperature, relative humidity and the masses measured before sample collection (M) in equation S1, m is found for each filter. After sample collection, a corrected mass for the filter is found from m, T and RH (again using equation S2.1) which is subtracted from the measured mass. This method will not account for changes in mass of the substance collected on the filter, which may have different hygroscopic properties to the filter.

**
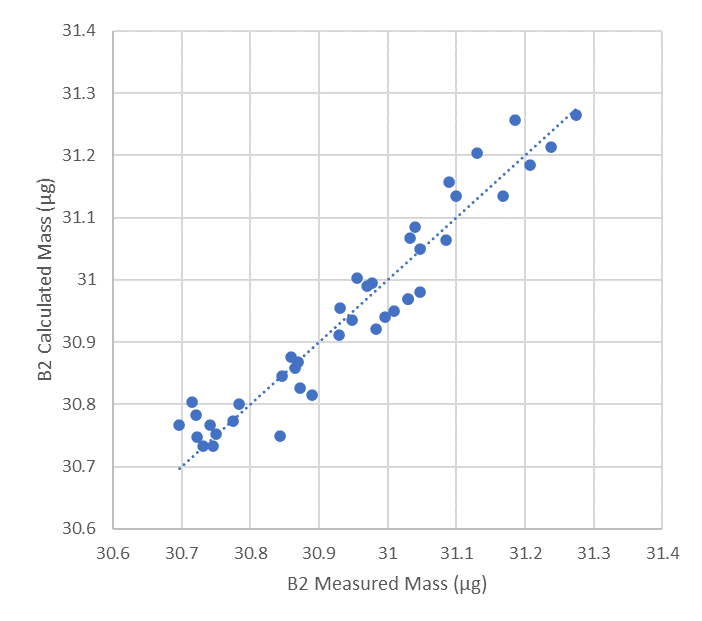
**

**Figure S2:** Calculated mass from temperature and humidity measurements in the weighing room against measured mass for filter blank B2.

Finally, a further correction for any other offsets within the weighing room was applied, if there was a difference between the corrected masses in the blanks measured before and after sampling, this was added as a linear offset to all filters. This was added to account for unidentified variables within the weighing room.

**Supplementary Materials 3: Aerosol mass, number and metal distributions**


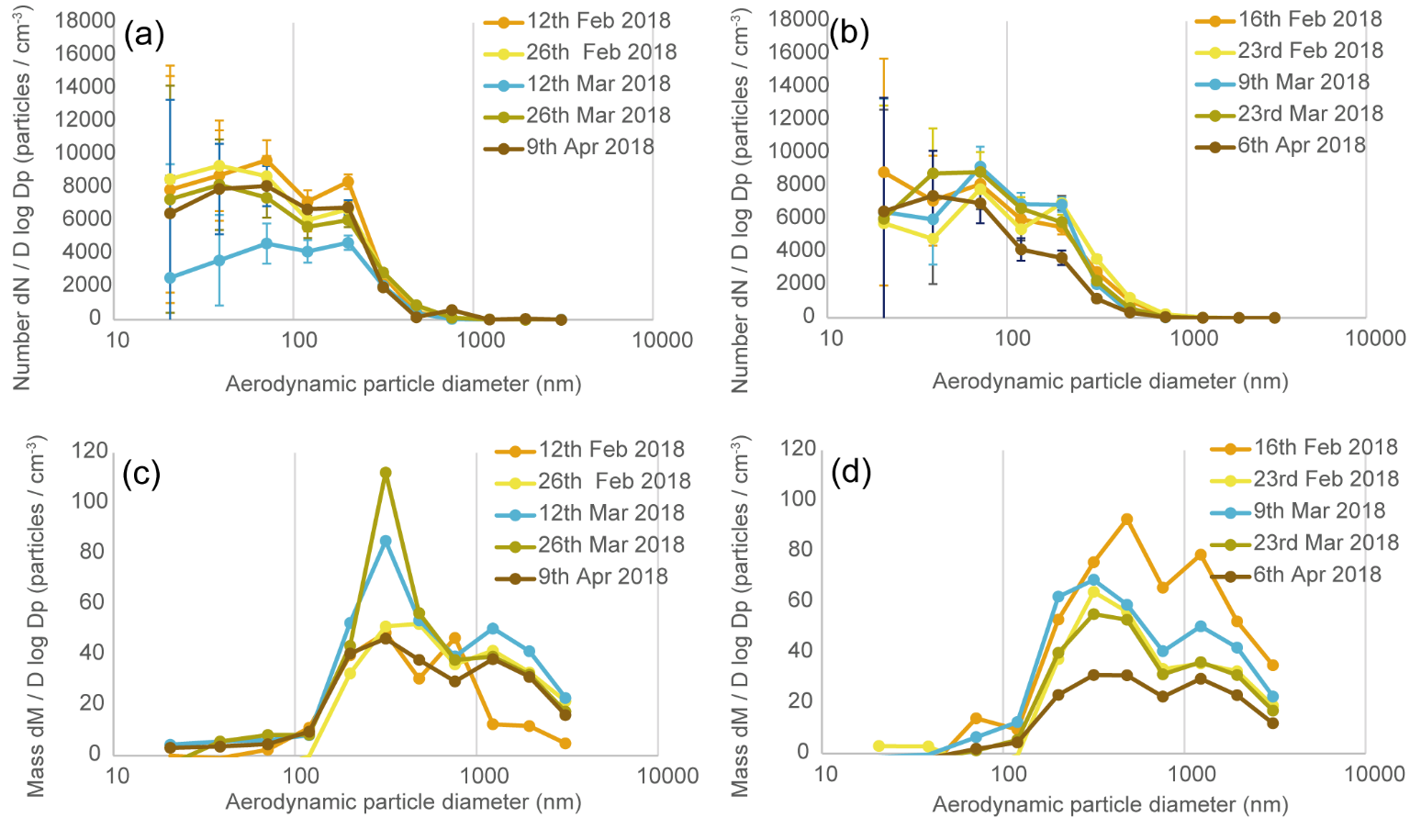


**Figure S3.** Number size distribution measured on each stage of the ELPI for (a) weekend and (b) weekday and mass size distribution measured on each stage of the ELPI for (c) weekend and (d) weekday, for ~ 3­‑day samples. Error bars in number size distribution represent drift in electrometers.


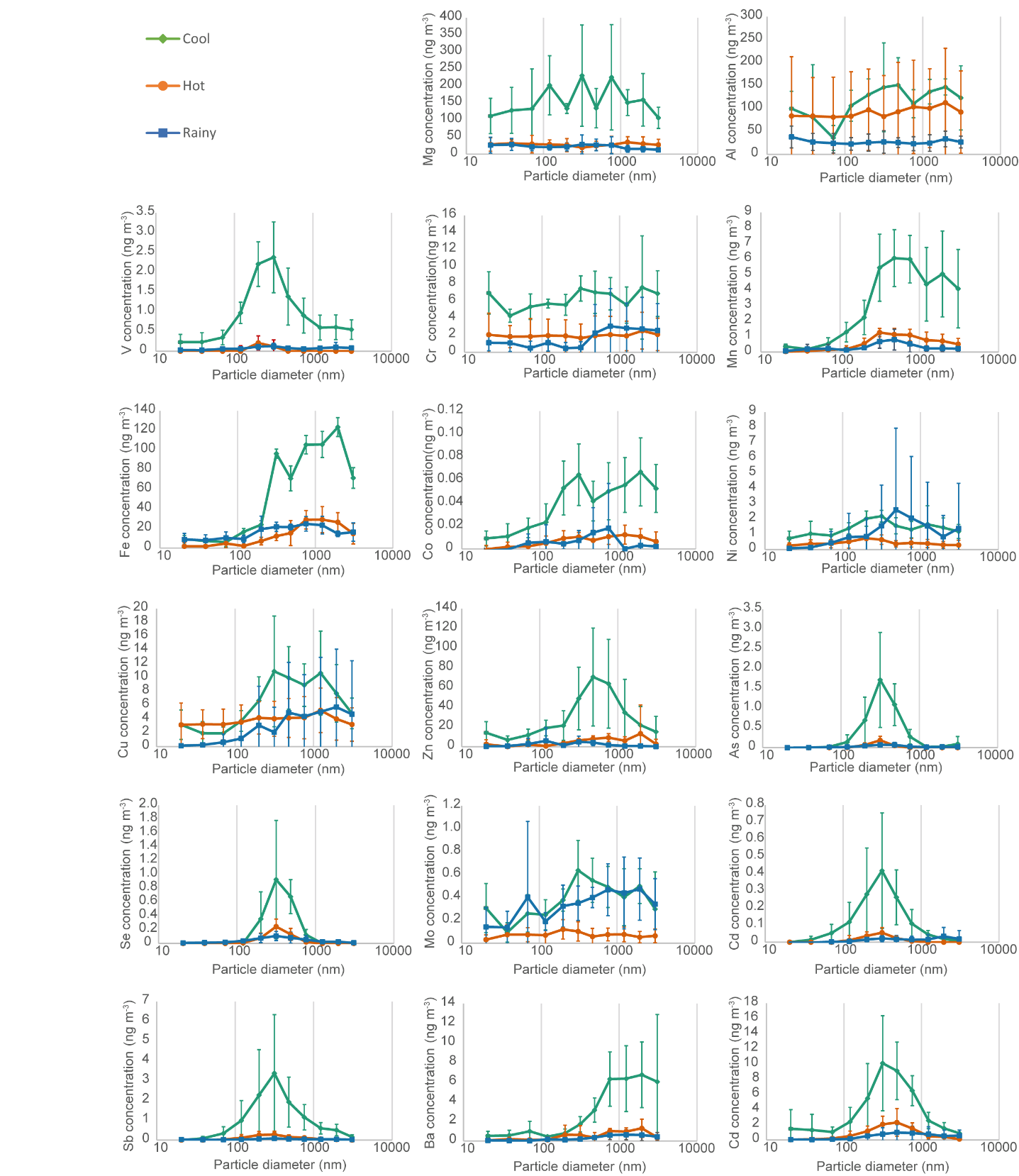


**Figure S4.** Airborne concentration of metal and metalloid within size separated samples of PM measured in near a toll road in Bangkok averaged over cool, hot and rainy seasons. Error bars show standard deviations. Data not normalised.


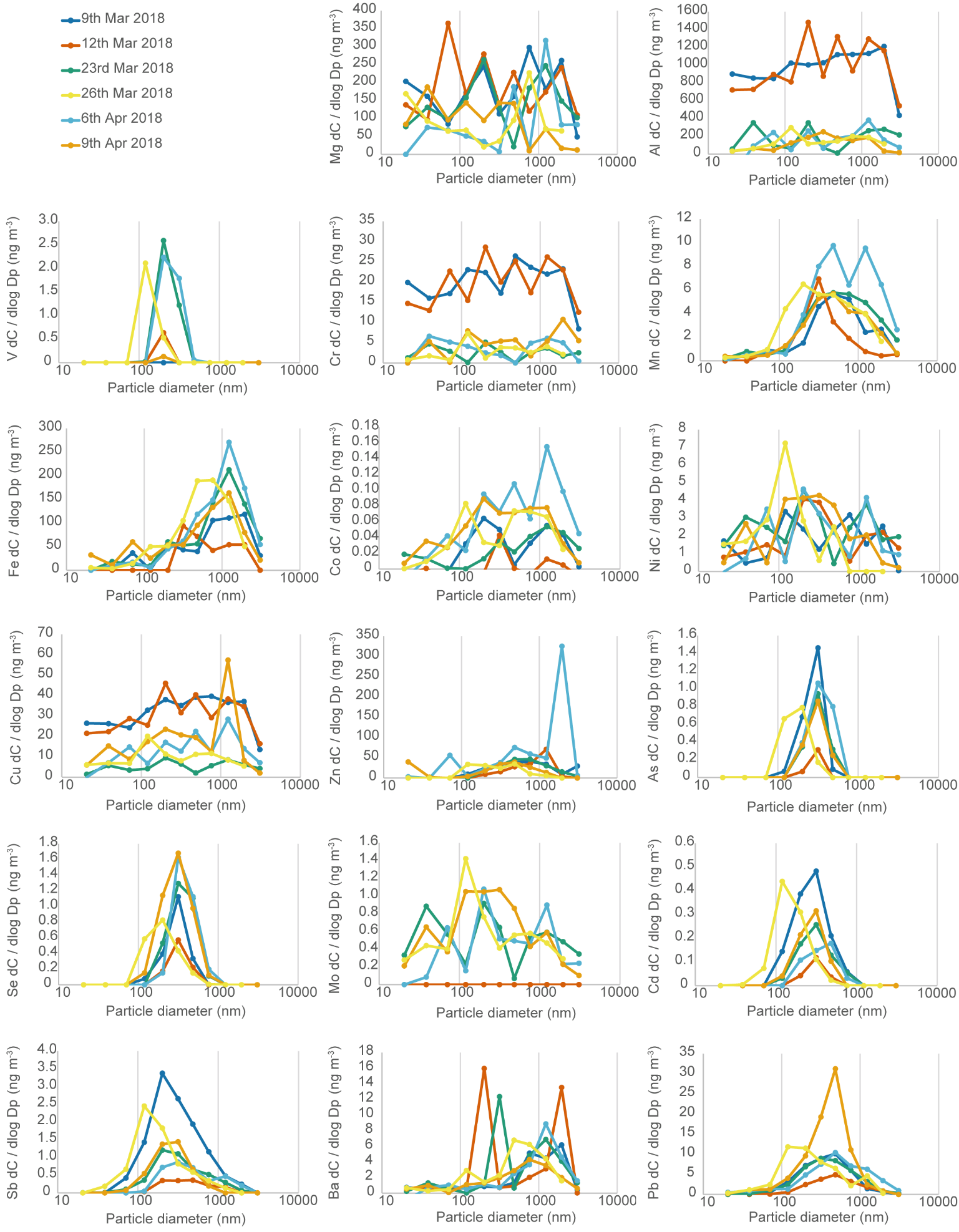


**Figure S5.** Airborne concentration of metal and metalloid within size separated sampled of PM measured in six 3-day samples near a toll road in Bangkok in hot season.


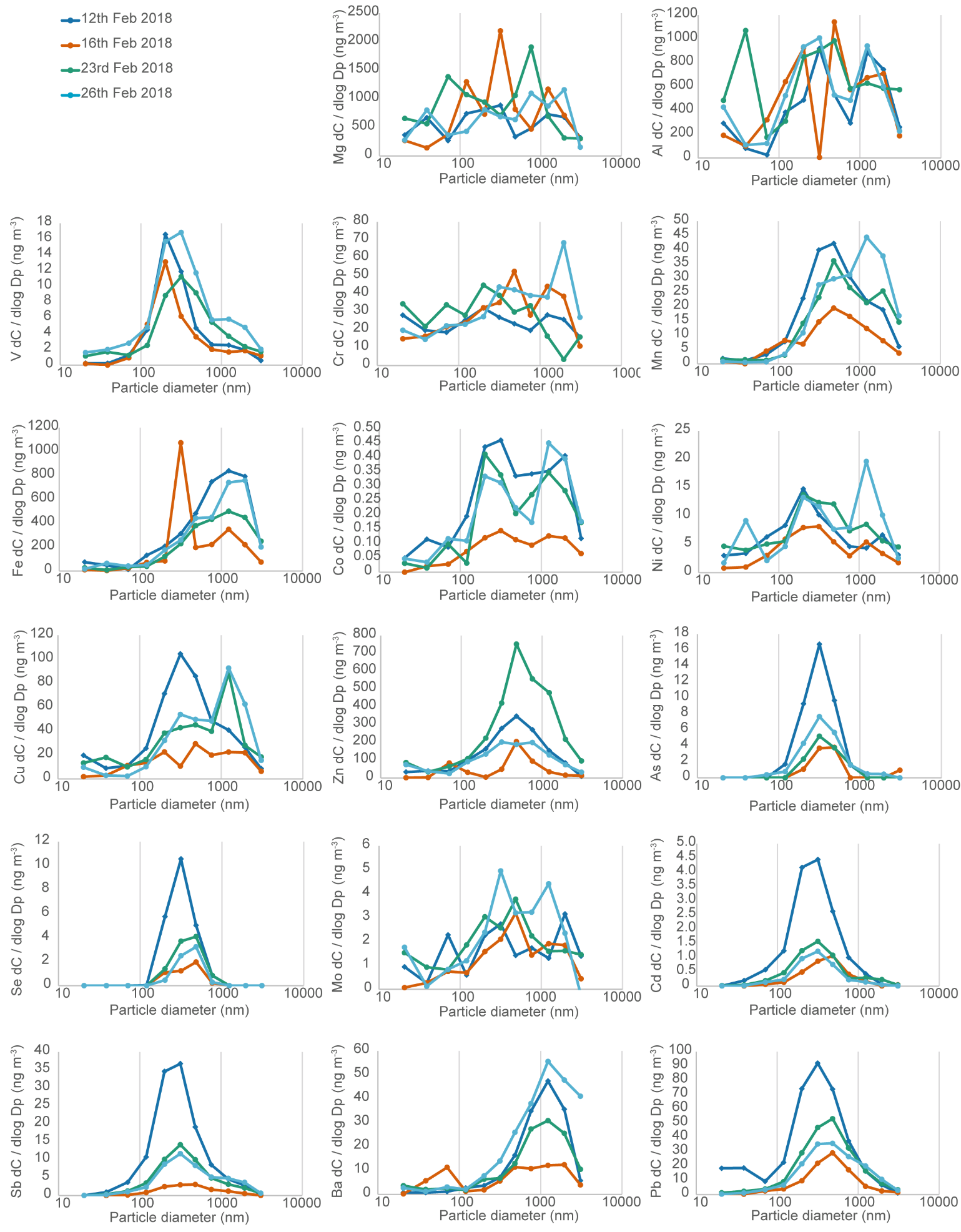


**Figure S6.** Airborne concentration of metal and metalloid within size separated sampled of PM measured in four 3-day samples near a toll road in Bangkok in cool season.


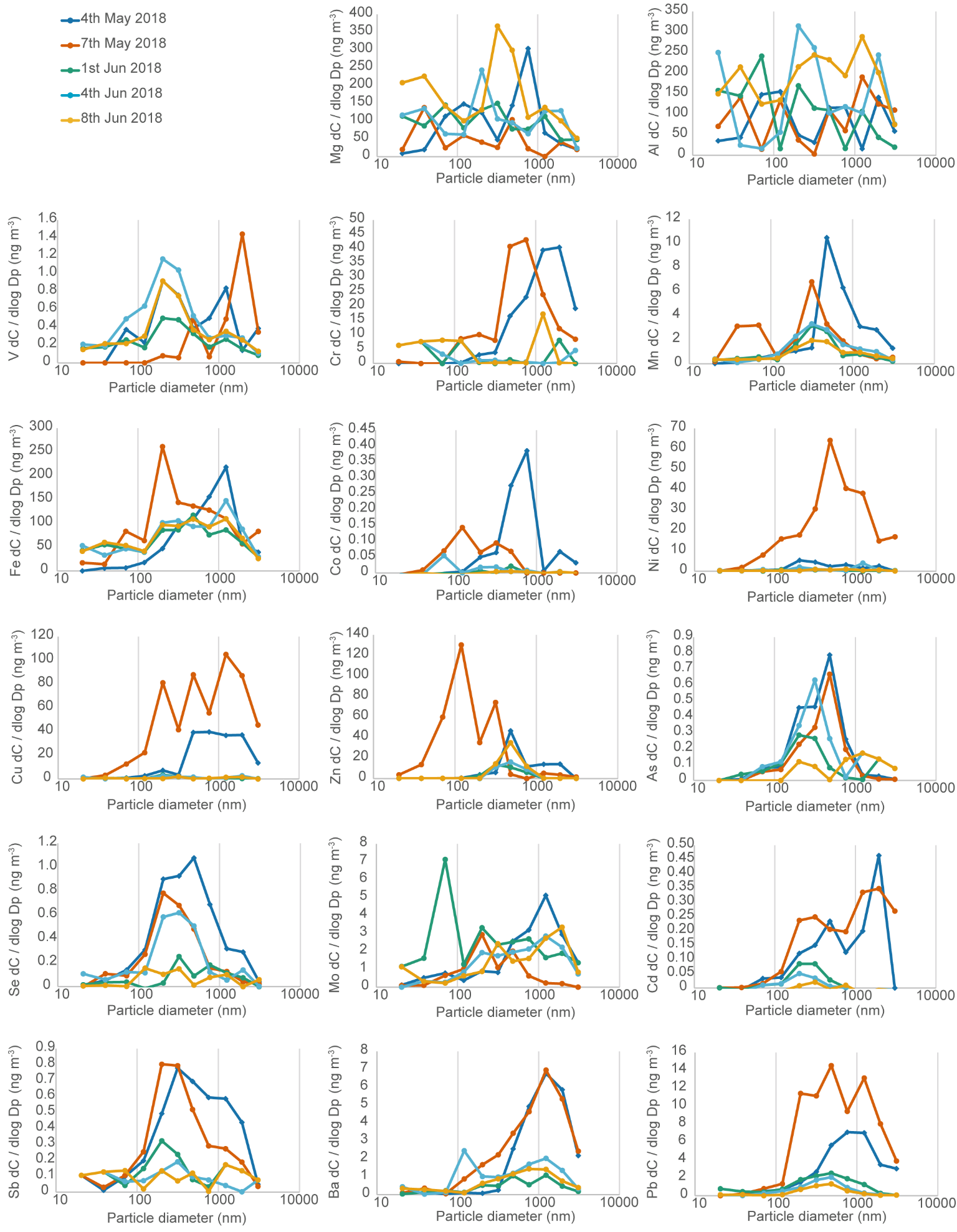


**Figure S7.** Airborne concentration of metal and metalloid within size separated sampled of PM measured in five 3-day samples near a toll road in Bangkok in rainy season.

**Supplementary Materials 4: Trajectory Analysis**

Biomass burning is a contributor to air quality in all SE asia, with crops burnt at specific times of the year. Peaks occur in February in Cambodia, Vietnam and PDR Laos (to the east of Bangkok) and in March in northern Thailand and Myanmar (to the north of Bangkok) (Vadrevu et al., 2019). Biomass burning in the northern Thailand is highest compared with other regions and the recent studies by Dejchanchaiwong et al. (2020) and Aman et al. (2020) showed that PM transported from biomass burning areas in northeast parts of Thailand, and some parts of Cambodia, Laos and Vietnam could contribute the increased PM levels in Bangkok.

We performed a trajectory analysis for the selected dates using Hybrid Single-Particle Lagrangian Integrated Trajectory Model (HYSPLIT) of the National Oceanic and Atmospheric Administration (NOAA) (Stein et al., 2015). The back trajectory integrated every hour for the selected dates and at 500 m AGL and migrates backward in time for 72 hours. HYSPLIT was run online at <http://www.ready.noaa.gov/HYSPLIT.php> using hourly 1 degree resolution global data assimilation system (GDAS) data for driving wind fields.

We chose three specific dates when measurements were taken in Bangkok: 26^th^ February, 27^th^ March and 9^th^ April, 2019. During these dates, the levels of PM were high in Bangkok and significant biomass burning may be occuring in nearby regions. Figures S7 to S8 show the trajectory analysis on these dates. These data show that air masses come from Laos and Vietnam during 9^th^ April, and from Cambodia during 26th February, but from the Ocean during 27th March.

The initial trajectory analysis during February-April has shown that the PM precursors emissions due to biomass burning could travel from the areas e.g. Cambodia, Laos, Vietnam and northern part of Thailand to Bangkok within these times, and may contrbute to the overall PM levels in the city.

**
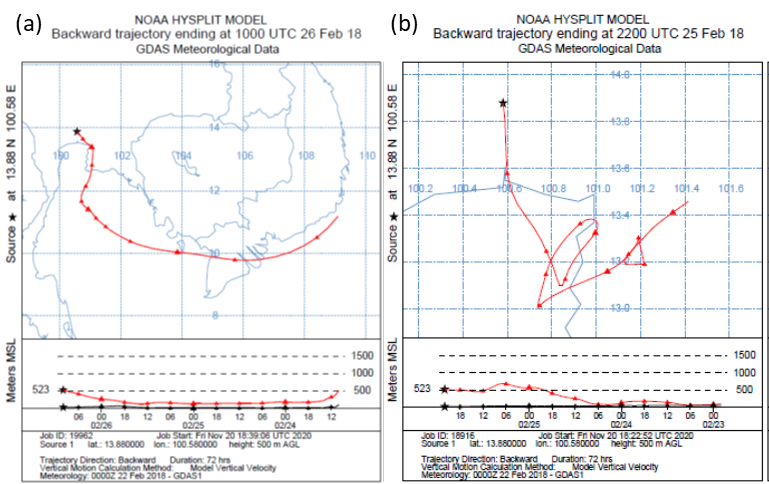
**

**Figure S8.** Calculated trajectory of air mass on 26^th^ February 2018 at (a) 1000 and (b) 2200 UTC

**
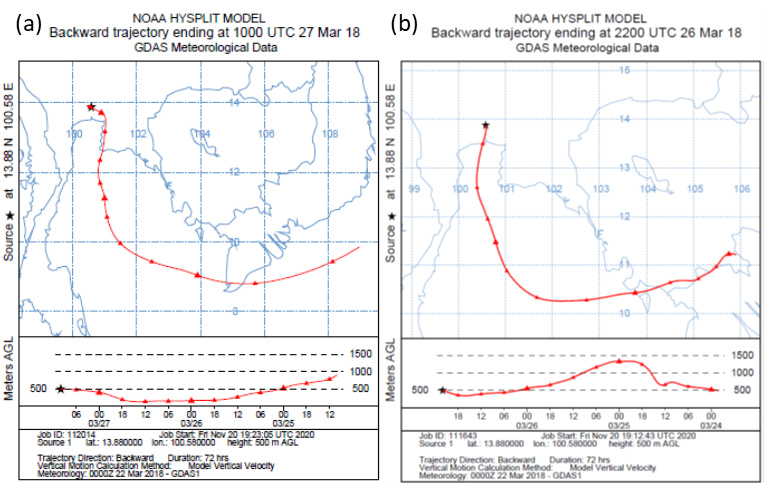
**

**Figure S9.** Calculated trajectory of air mass on 27^th^ March 2018 at (a) 1000 and (b) 2200 UTC


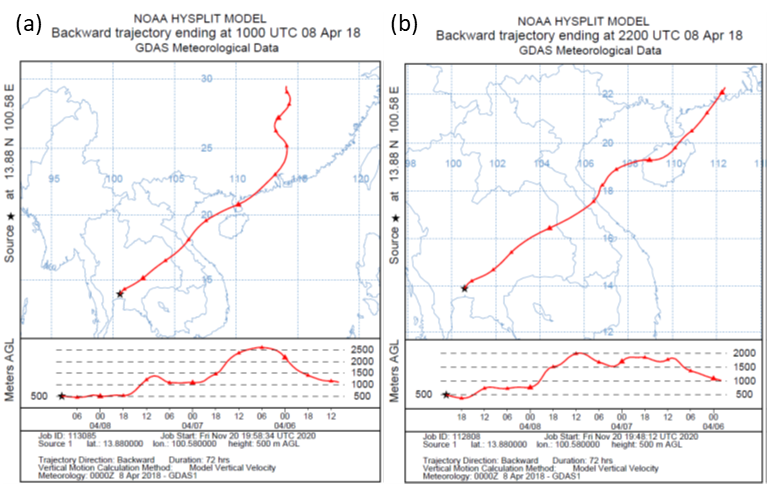


**Figure S10** Calculated trajectory of air mass on 9^th^ April 2018 at (a) 1000 and (b) 2200 UTC

**Supplementary Materials 5: Principal Components**

Within the analysis, we display the first 5 principal components (PC), but 18 were present in the analysis. The results show that the first PC, which is well correlated with total mass of the particles, explains almost half of the variance, and the first five PCs account for 78% of the variance. Each of the remaining PCs accounts for just a few percent of the remaining variance and so were omitted from the analysis.

**
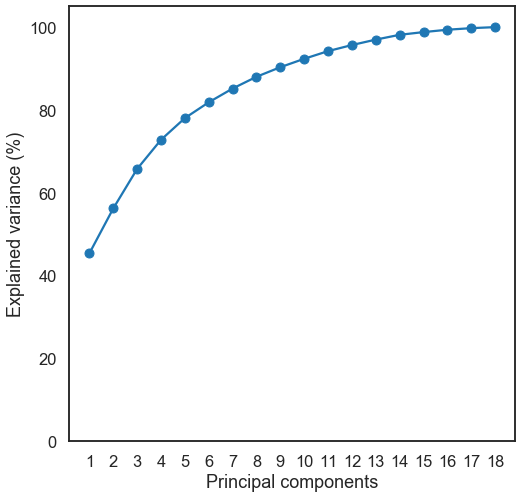
**

**Figure S11**: Explained variance of PM_10_ data from principal component analysis of metal concentrations.

**Supplementary Materials 6: Health Risk Assessment**

***Health Risk Analysis***

Health risk of exposure to trace metals bound to atmospheric particles was assessments according to methodology developed by US Environmental Protection Agency (US-EPA). In this study, health risk assessments of metals were conducted for carcinogenic and non-carcinogenic risks from inhalation and ingestion (1, 2). The exposure concentration from inhalation (EC_inhalation_) and ingestion determined by average daily dose (ADD_ingestion_) were calculated according to Eqs 1 and 2, respectively.

The exposure concentration for inhalation (EC_inhalation_) is calculated according to Eq S2

$\mathrm{EC}_{\mathrm{inhalation}}=\frac{C\times ET\times EF\times ED}{\mathrm{AT}}$ (S2)

Where C refers to the concentrations of trace elements in ambient air (μg/m^3^) which is the average concentrations from dry and winter seasons representing the exposure level throughout the year, ET is exposure time (ET = 24 hours/day); EF is exposure frequency (EF = 365 days/year); ED is exposure duration (ED = 24 years for adults); AT is the average time (AT = ED × 365 days for non-carcinogens, AT = 70 × 365 days for carcinogens.

The exposure concentration for ingestion determined by average daily dose (ADD_ingestion_) is calculated according to Eq S3

$\mathrm{ADD}_{\mathrm{ingestion}}=\frac{C\times IngR\times EF\times ED}{BW\times AT}$ (S3)

Where C refers to the concentrations of trace elements (µg kg^-1^), IngR is the ingestion rate of 100 mg day^-1^ for adults, EF is exposure frequency (EF = 365 days/year), ED is exposure duration (ED = 24 years for adults), BW is the average body weight (BW = 70 kg) and AT = 70 × 365 days for carcinogens.

The health risks were characterized by non-carcinogenic and carcinogenic effects using equations of hazard quotient (HQ) and cancer risk (CR), respectively (Eqs S4 and S5).

$$HQ={\mathrm{EC}_{\mathrm{inhalation}}}/\mathrm{RfCi}$$

$={\mathrm{ADD}_{\mathrm{ingestion}}}/\mathrm{RfDo}$ (S4)

$$CR=\mathrm{EC}_{\mathrm{inhalation}}\times IUR$$

$=\mathrm{ADD}_{\mathrm{ingestion}}\times SFo$ (S5)

Where, RfCi is chronic inhalation reference concentration (mg m^-3^), RfDo is chronic oral reference dose (mg/kg/day), IUR is inhalation unit risk (μg m^-3^)^-1^ and SFo is oral slope factor (mg/kg/day)^-1^(5).

HQ is used for assessing the level of concern for each non-carcinogenic metal, and the hazard index (HI) is the sum of the HQs for several chemicals. The HQ or HI >1 suggests that adverse health effects are likely to occur. Cancer risk (CR) was used to assess carcinogenic risk, and the tolerable value is 10^-6^ to 10^-4^, while >10^-4^ indicates that the risk exceeds the acceptable level (4).

**PM_10_**

Figure S10(a) and S10(b) present the HQ values of particulate bound trace elements by inhalation and ingestion exposure, respectively. HI values (sum of HQ values for all elements)

*Non carcinogenic health risk assessment*


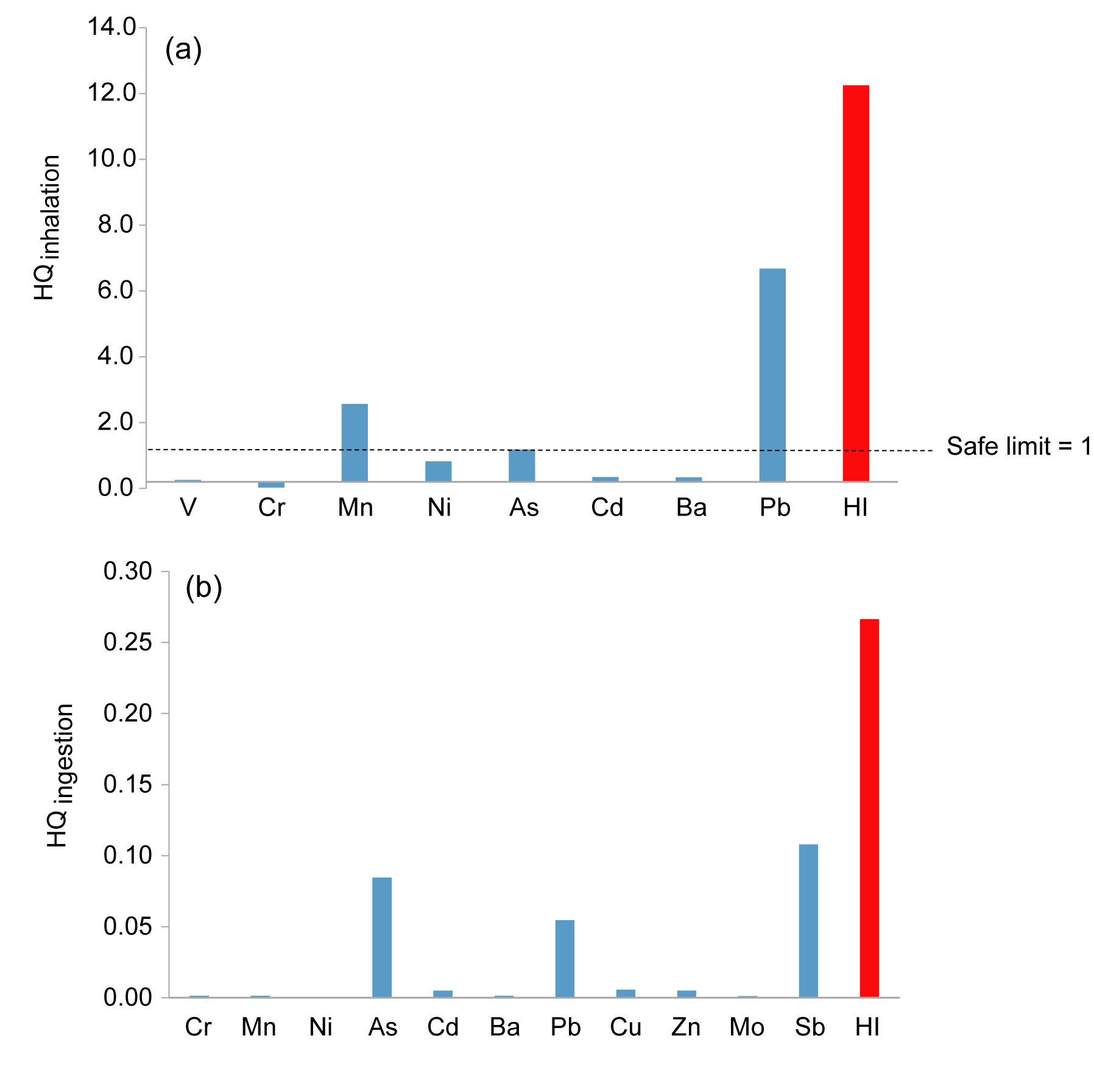


**Fig S12** Non-carcinogenic risk of exposure to PM_10_ via inhalation (a) and ingestion (b)

*Carcinogenic health risk assessment*

The CR value between 10^-6^ and 10^-4^ is considered to pose a potential cancer risk and CR values with
 > 10^-4^ are considered to highly likely cause cancer.

*
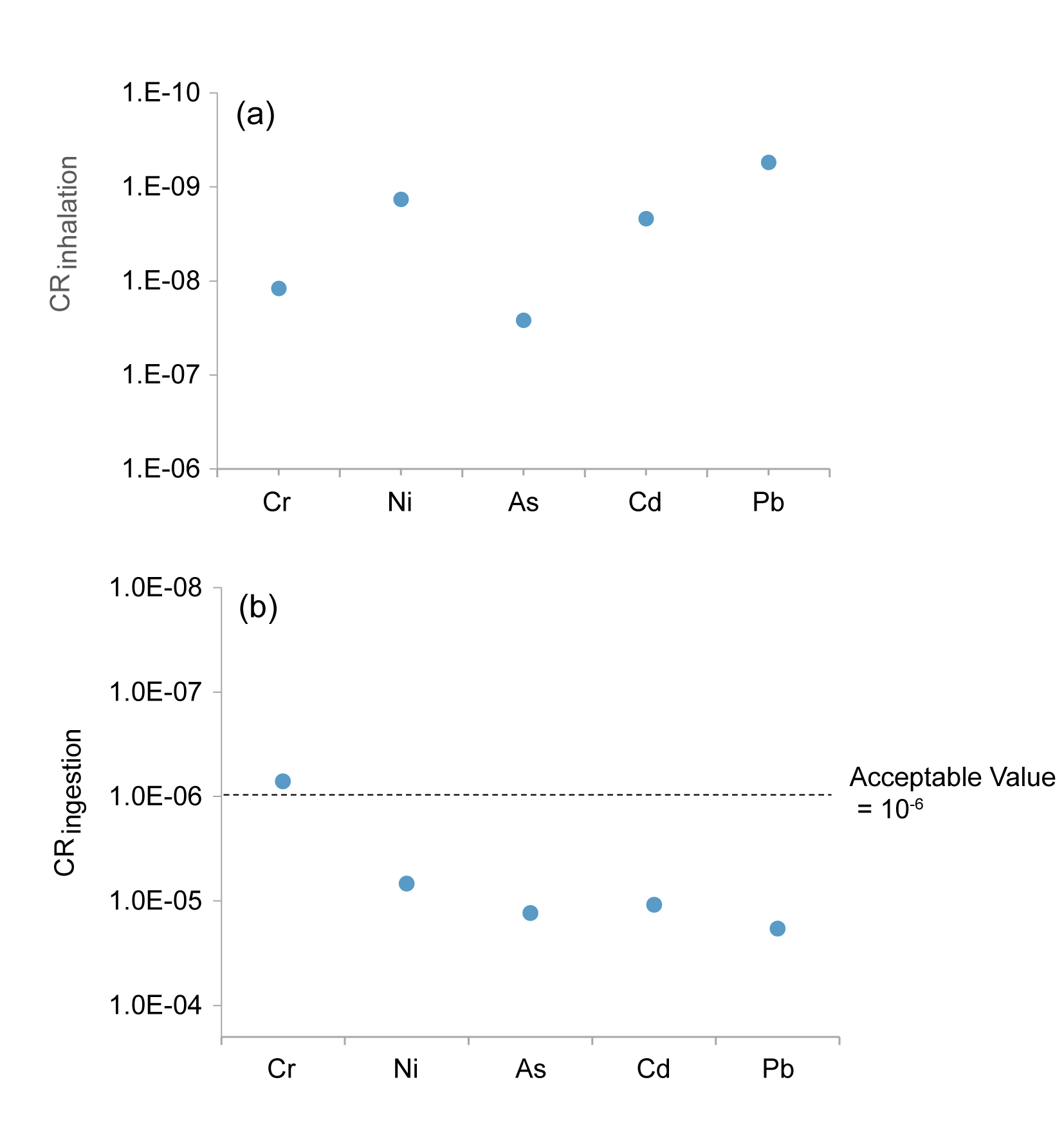
*

**Fig S13** Carcinogenic risk of exposure to PM_10_ via inhalation (a) and ingestion (b)

**Size distribution**

*Non carcinogenic health risk assessment*

**
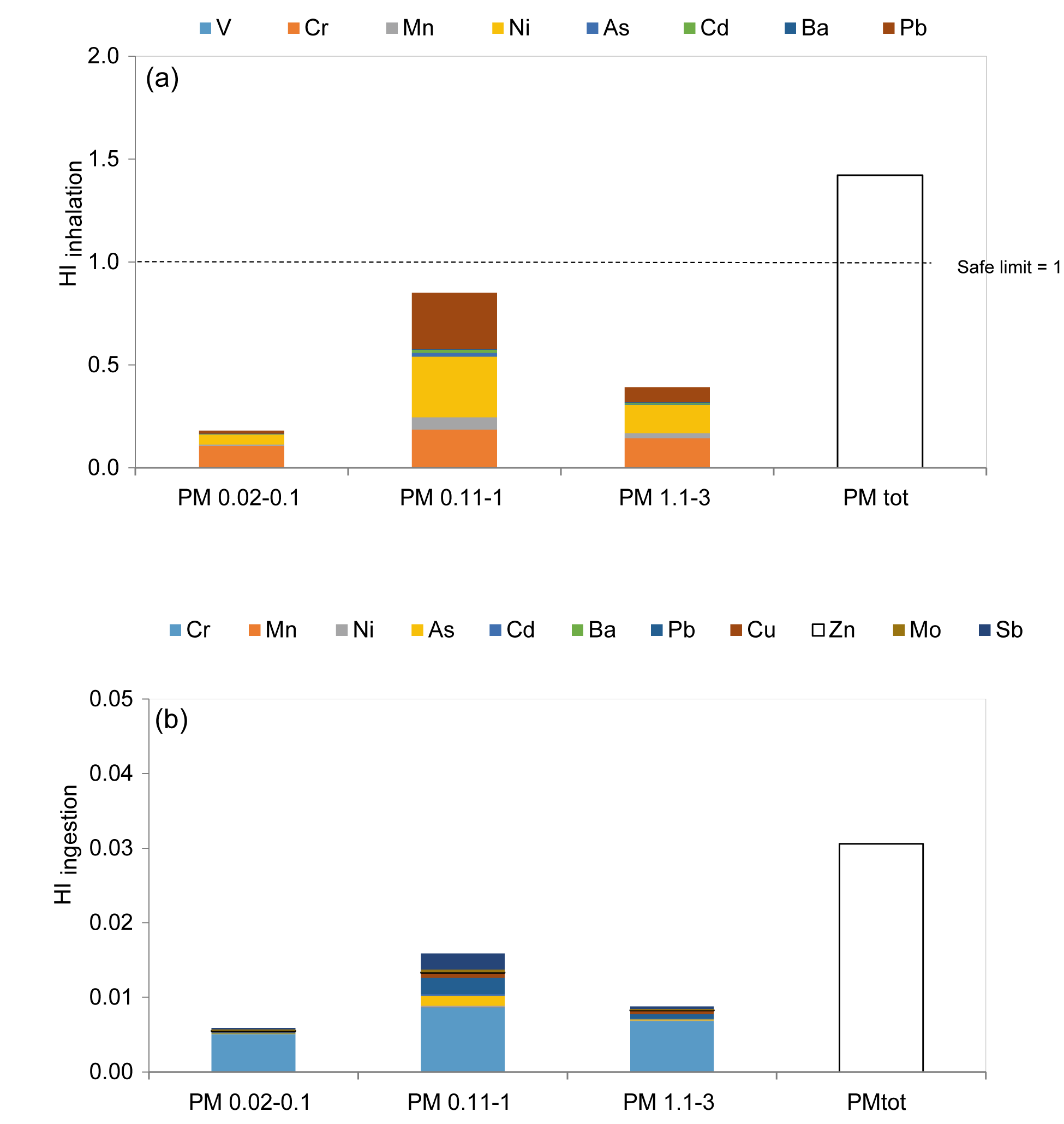
**

**Fig S14** Non-carcinogenic risk of exposure to metals bound to various size of PM via inhalation (a) and ingestion (b)

*Carcinogenic health risk assessment*


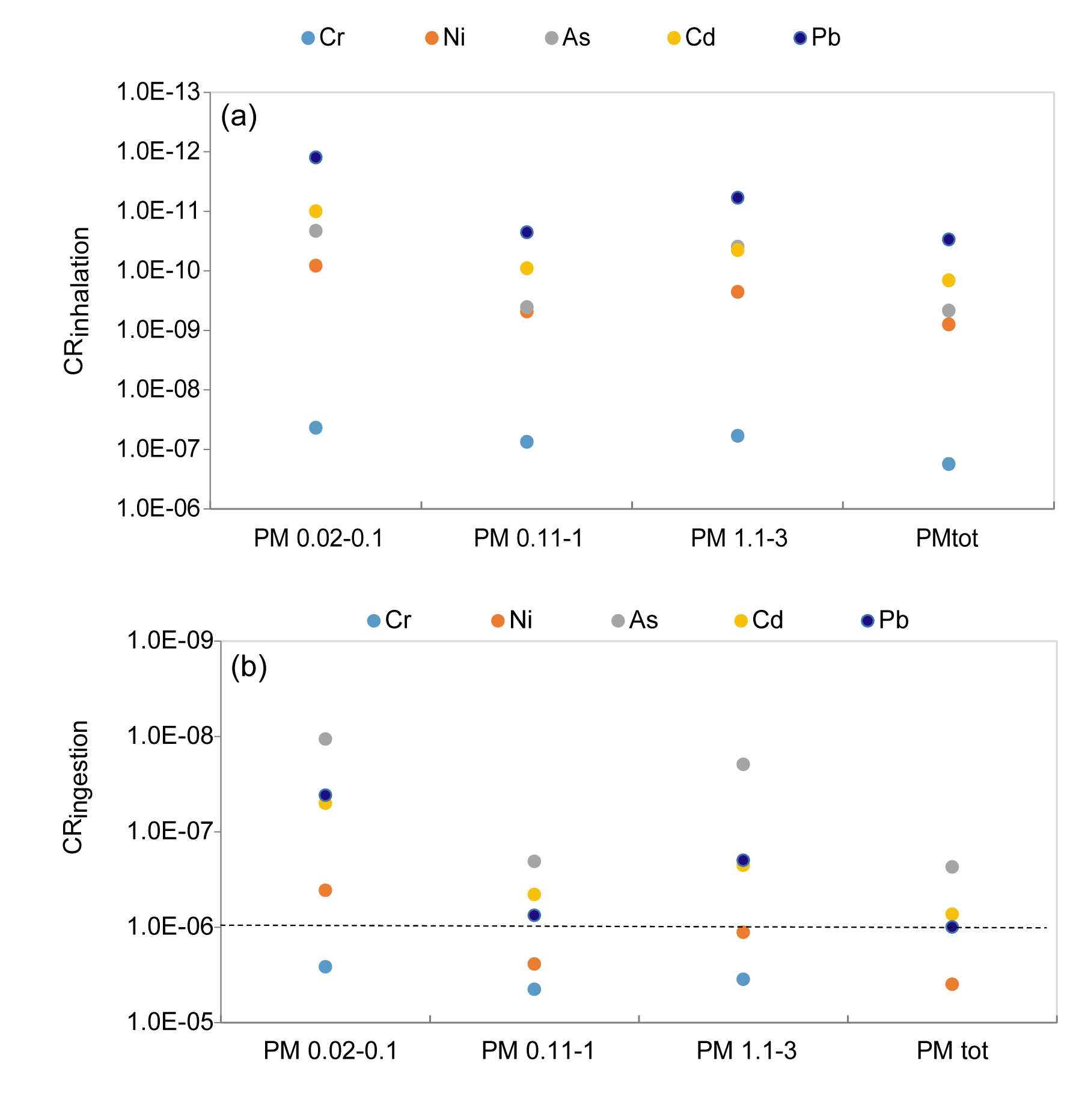


**Fig S15** Carcinogenic risk of exposure to metals bound to various size of PM via inhalation (a) and ingestion (b)

**Table S3** Carcinogenic and non-carcinogenic risks of each element via inhalation and ingestion exposure

| **Elements** | **Inhalation exposure** | |  |  |  | **Ingestion exposure** | |  |  |
| --- | --- | --- | --- | --- | --- | --- | --- | --- | --- |
|  | **PM_0.02-0.1_** | **PM_0.11-1_** | **PM_1.1-3_** | **PM_10_** |  | **PM_0.02-0.1_** | **PM_0.11-1_** | **PM_1.1-3_** | **PM_10_** |
|  | **Carcinogenic (CR)** | |  |  |  |  |  |  |  |
| **Cr** | 4.4E-08 | 7.5E-08 | 5.9E-08 | 1.2E-08 |  | 2.6E-06 | 4.5E-06 | 3.5E-06 | 7.1E-07 |
| **Ni** | 8.2E-11 | 4.8E-10 | 2.3E-10 | 1.4E-09 |  | 4.1E-07 | 2.4E-06 | 1.1E-06 | 6.8E-06 |
| **As** | 2.1E-11 | 4.1E-10 | 3.9E-11 | 2.6E-08 |  | 1.1E-08 | 2.0E-07 | 2.0E-08 | 1.3E-05 |
| **Cd** | 1.0E-11 | 9.1E-11 | 4.5E-11 | 2.2E-09 |  | 5.0E-08 | 4.5E-07 | 2.2E-07 | 1.1E-05 |
| **Pb** | 1.2E-12 | 2.2E-11 | 5.9E-12 | 5.5E-10 |  | 4.1E-08 | 7.5E-07 | 2.0E-07 | 1.8E-05 |
|  | **Non-Carcinogenic (HQ)** | | |  |  |  |  |  |  |
| **Cr** | 1.1E-01 | 1.8E-01 | 1.4E-01 | 2.9E-02 |  | 5.0E-03 | 8.7E-03 | 6.8E-03 | 1.4E-03 |
| **Mn** | 6.0E-03 | 5.9E-02 | 2.5E-02 | 2.6E+00 |  | 3.1E-06 | 3.0E-05 | 1.3E-05 | 1.3E-03 |
| **Ni** | 5.0E-02 | 2.9E-01 | 1.4E-01 | 8.3E-01 |  | 2.8E-05 | 1.7E-04 | 7.8E-05 | 4.7E-04 |
| **As** | 9.6E-04 | 1.8E-02 | 1.8E-03 | 1.2E+00 |  | 6.9E-05 | 1.3E-03 | 1.3E-04 | 8.4E-02 |
| **Cd** | 1.6E-03 | 1.5E-02 | 7.3E-03 | 3.5E-01 |  | 2.3E-05 | 2.1E-04 | 1.0E-04 | 5.0E-03 |
| **Ba** | 6.2E-04 | 4.2E-03 | 4.3E-03 | 3.4E-01 |  | 2.2E-06 | 1.5E-05 | 1.5E-05 | 1.2E-03 |
| **Pb** | 1.5E-02 | 2.7E-01 | 7.2E-02 | 6.7E+00 |  | 1.2E-04 | 2.2E-03 | 5.9E-04 | 5.5E-02 |
| **V** | 8.7E-04 | 4.3E-03 | 8.1E-04 | 2.6E-01 |  |  |  |  |  |
| **Cu** |  |  |  |  |  | 2.0E-04 | 6.2E-04 | 4.8E-04 | 5.5E-03 |
| **Zn** |  |  |  |  |  | 2.2E-05 | 9.9E-05 | 5.3E-05 | 5.0E-03 |
| **Mo** |  |  |  |  |  | 2.1E-04 | 3.5E-04 | 1.8E-04 | 1.0E-03 |
| **Sb** |  |  |  |  |  | 1.8E-04 | 2.2E-03 | 3.3E-04 | 1.1E-01 |
| **HI** | **1.8E-01** | **8.5E-01** | **3.9E-01** | **1.2E+01** |  | **5.9E-03** | **1.6E-02** | **8.8E-03** | **2.7E-01** |

Acceptable value of carcinogenic and non-carcinogenic were CR 10^-6^ to 10^-4^ and HQ or HI > 1, respectively

**Table S4** Exposure factors for health risk assessment model

| **Parameters** | **Definition** | **Values** |
| --- | --- | --- |
| **C** (μg/m^3^) | Concentration of elements | measurement |
| **ET**  (hrs) | Exposure Time | 24 |
| **EF** (days/yesr) | Exposure Frequency | 365 |
| **ED** for adult (years) | Exposure Duration | 24 |
| **AT**(day) | Average Exposure Time |  |
| - non-carcinogens |  | ED×365 days |
| - carcinogens |  | 70×365days |
| **BW** (kgs) | Body Weight | 70 |

**Table S5** The values of RfD, IUR and SFo for elements

| **Parameters** | **Cr** | **Ni** | **As** | **Cd** | **Pb** | **Ba** | **Mn** | **Cu** | **Zn** | **Mo** | **Sb** | **V** |
| --- | --- | --- | --- | --- | --- | --- | --- | --- | --- | --- | --- | --- |
| **RfD;** reference dose |  |  |  |  |  |  |  |  |  |  |  |  |
| - Inhalation (mg/kg/day) | 1.0E-04 | 2.0E-05 | 1.50E-05 | 1.0E-05 | 2.0E-05 | 5.0E-04 | 5.0E-05 |  |  |  |  | 1.0E-04 |
| - Ingestion (mg/kg/day) | 3.0E-03 | 5.0E-02 | 3.0E-04 | 1.0E-03 | 3.5E-03 | 2.0E-01 | 1.4E-01 | 4.0E-02 | 3.0E-01 | 5.0E-03 | 4.0E-04 |  |
| **IUR;** Inhalation unit risk | 1.2E-02 | 2.4E-04 | 4.3E-03 | 1.8E-03 | 1.2E-05 |  |  |  |  |  |  |  |
| (µg/m^3^) |  |  |  |  |  |  |  |  |  |  |  |  |
| **SFo**; Oral slope factor | 5.0E-01 | 8.4E-01 | 1.5E+00 | 6.3E+00 | 2.8E-01 |  |  |  |  |  |  |  |
| (mg/kg/day) |  |  |  |  |  |  |  |  |  |  |  |  |

**References**

EPA U. Risk Assessment Guidance for Superfund Volume I: Human Health Evaluation Manual (Part a). 1989.

EPA U. Risk Assessment Guidance for Superfund Volume I: Human Health Evaluation Manual. Part F: Supplemental Guidance for Inhalation Risk Assessment. 2009.

EPA US. Regional Screening Levels (RSLs)—Generic Tables. [Accessed: 2019 July 3]. 2019.

Liu K, Shang Q, Wan C. Sources and health risks of heavy metals in PM2. 5 in a campus in a typical suburb area of Taiyuan, North China Atmosphere 2018; 9(2):46.

Zhou X, Strezov V, Jiang Y, Yang X, Kan T, Evans T. Contamination identification, source apportionment and health risk assessment of trace elements at different fractions of atmospheric particles at iron and steelmaking areas in China. PloS one 2020; 15(4):e0230983.

1. * Corresponding author. Tel: +44 117 93 88765

   E*-mail address*: j.c.matthews@bristol.ac.uk [↑](#footnote-ref-1)
